# An ^211^At-labeled alpha-melanocyte stimulating hormone peptide analog for targeted alpha therapy of metastatic melanoma

**DOI:** 10.1007/s00259-024-07056-3

**Published:** 2025-01-20

**Authors:** Hiroyuki Suzuki, Saki Yamashita, Shoko Tanaka, Kento Kannaka, Ichiro Sasaki, Yasuhiro Ohshima, Shigeki Watanabe, Kazuhiro Ooe, Tadashi Watabe, Noriko S. Ishioka, Hiroshi Tanaka, Tomoya Uehara

**Affiliations:** 1 Graduate School of Pharmaceutical Sciences, Chiba University, 1-8-1 Inohana, Chuo-ku, Chiba 260-8675 Japan; 2 Department of Quantum-Applied Biosciences, Takasaki Institute for Advanced Quantum Science, National Institutes for Quantum Science and Technology, Watanuki, Takasaki, 1233, 370-1292 Gunma Japan; 3 Radioisotope Research Center, Institute for Radiation Sciences, Osaka University, 2-4 Yamadaoka, Suita, Osaka, 565-0871 Japan; 4 Department of Radiology, Graduate School of Medicine, Osaka University, 2-2 Yamadaoka, Suita, 565-0871 Osaka Japan; 5 Faculty of Pharmacy, Juntendo University, 6-8-1 Hinode, Urayasu, 279-0013 Chiba Japan; 6 Department of Chemical Science and Engineering, Institute of Science Tokyo, 2-12-1 Ookayama, Meguro-ku, Tokyo, 152-8552 Japan

**Keywords:** Targeted alpha therapy, ^211^At, Metastatic melanoma, Melanocortin-1 receptor, Alpha-melanocyte stimulating hormone

## Abstract

**Purpose:**

Patients who develop metastatic melanoma have a very poor prognosis, and new treatments are needed to improve the response rates. Melanocortin-1 receptor (MC1R) is a promising target for radionuclide therapy of metastatic melanoma, and alpha-melanocyte stimulating hormone (α-MSH) peptide analogs show high affinities to MC1Rs. Because targeted alpha therapy (TAT) can be a desirable treatment for metastatic melanoma, this study aimed to develop an ^211^At-labeled α-MSH peptide analog for TAT of metastatic melanoma.

**Methods:**

We designed an α-MSH analog labeled with ^211^At using a neopentyl glycol scaffold via a hydrophilic linker. Preliminary studies using ^125^I-labeled α-MSH analogs were performed to identify suitable hydrophilic linkers. Then, [^211^At]NpG-GGN**4c** was prepared using a procedure similar to that of the ^125^I-labeled counterpart, [^125^I]NpG-GGN**4b**. The biodistribution profile of [^211^At]NpG-GGN**4c** in B16F10 tumor-bearing mice was compared with that of [^125^I]NpG-GGN**4b**. B16F10 tumor-bearing mice were treated with a single dose of vehicle or [^211^At]NpG-GGN**4c** (1 or 0.4 MBq**)**.

**Results:**

The D-Glu-D-Arg linker was identified as the optimal hydrophilic linker because of its high affinity for MC1R and good biodistribution profile, especially with low accumulation in the liver and intestine. [^211^At]NpG-GGN**4c** showed tumor accumulation comparable to that of [^125^I]NpG-GGN**4b** and maintained the tumor radioactivity retention from 1 to 3 h postinjection. [^211^At]NpG-GGN**4c** exhibited a dose-dependent inhibitory effect on B16F10 xenograft growth without apparent body weight loss.

**Conclusion:**

[^211^At]NpG-GGN**4c** showed dose-dependent efficacy against B16F10 xenografts, suggesting that [^211^At]NpG-GGN**4c** is a promising TAT agent for treating metastatic melanoma.

**Supplementary Information:**

The online version contains supplementary material available at 10.1007/s00259-024-07056-3.

## Introduction

Malignant melanoma is the most aggressive form of skin cancer, with an increasing incidence. Recent advances in the treatment of malignant melanoma, such as B-Raf proto-oncogene serine/threonine kinase (BRAF) and mitogen-activated extracellular signal-related kinase (MEK) inhibitors and immune checkpoint inhibitors, have improved the overall survival of the patients [1, 2]. However, the response rates to these treatments are limited. Metastatic melanoma is extremely aggressive and associated with high mortality. Therefore, new treatments for metastatic melanoma with improved response rates are needed. Radionuclide therapy using β-emitting radionuclides was less effective against metastatic melanoma, due to the low linear energy transfer (LET) and long path length [3]. Whereas, targeted alpha therapy (TAT) can be a desirable treatment for metastatic melanoma owing to the high LET and short path length of alpha particles [4, 5]. Among clinically applicable alpha emitters, ^225^Ac is currently regarded as the most promising alpha-emitters for TAT, but its limited global supply is a problem [6]. Whereas, ^211^At can be produced in a cyclotron using a well-established protocol [7]. Furthermore, the absence of long-lived daughter alpha emitters among the simple decays of ^211^At would be advantageous for avoiding side effects.

Alpha-melanocyte stimulating hormone (α-MSH) peptide analogs show high affinities to melanocortin-1 receptors (MC1Rs) that are overexpressed on melanoma tumor cells [8]. Rapid accumulation in the targeted cancer tissue is desired for the delivery of ^211^At because of its relatively short half-life (7.2 h), and thus α-MSH analogs are attractive vehicles for the delivery of ^211^At to metastatic melanoma. In this study, we aimed to develop ^211^At-labeled α-MSH peptide analogs for the treatment of metastatic melanoma. Although various radiolabeled α-MSH analogs have been developed, ^211^At-labeled α-MSH analogs have not been reported, and even radioiodinated analogs are limited for a few [9]. We referred to a representative α-MSH analog, DOTA-GGNle-CycMSH_hex_, which is used for labeling with radiometals such as ^111^In, ^68^Ga, ^90^Y, ^177^Lu, and ^203^Pb [10]. The high hydrophilicity of DOTA acts as a pharmacokinetic modifier and contributes to low accumulation in the liver and intestine and increases urinary excretion of many radiometal-labeled peptides [11]. In general, radiolabeled peptides with low hydrophilicity are eliminated from the body via the hepatobiliary route, causing rapid and high accumulation in the liver and intestine [12]. According to the previous studies, accumulation in the liver and intestine impairs tumor accumulation of radiolabeled α-MSH analogs [13, 14].

**Fig. 1:**
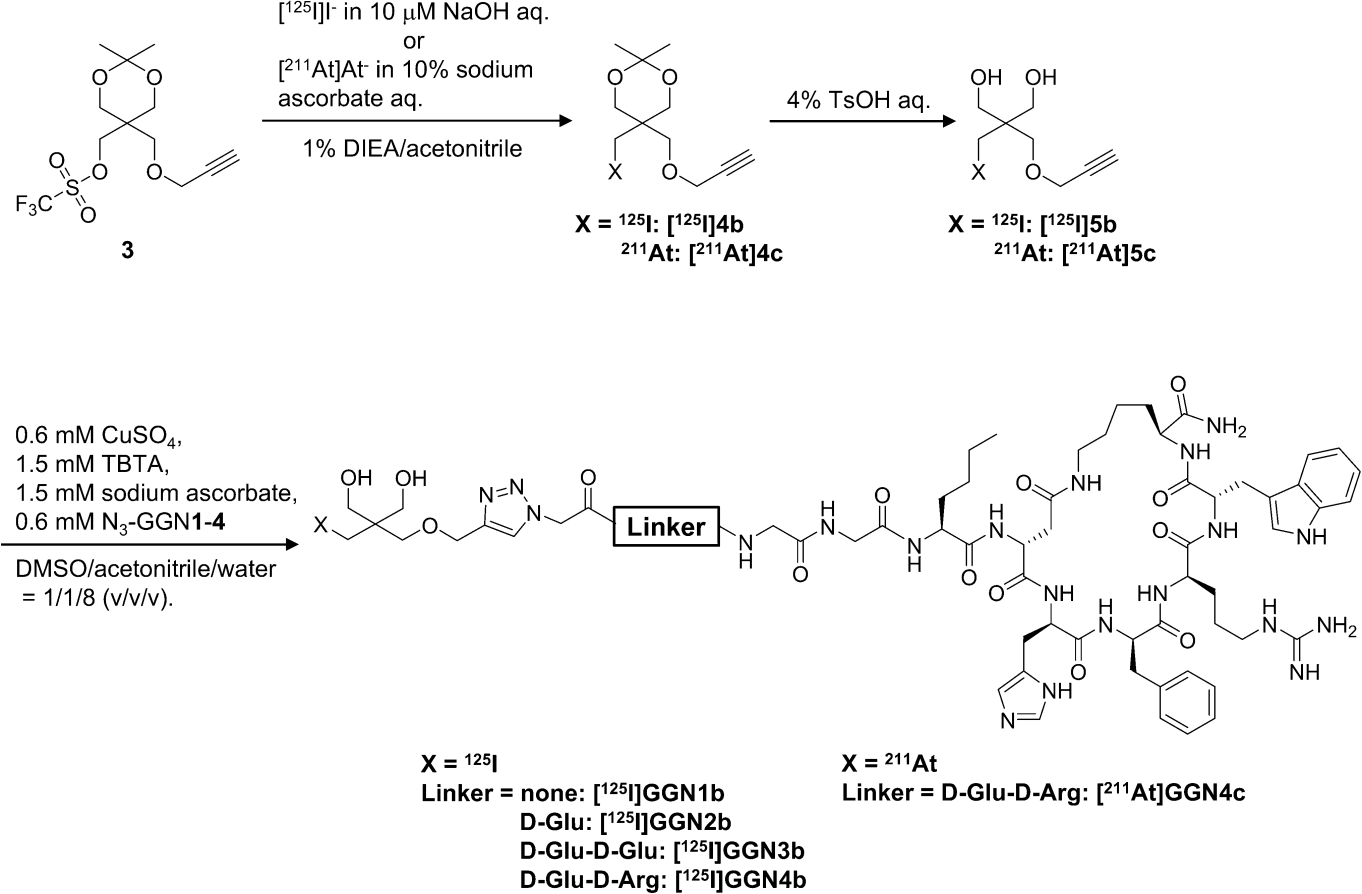
Synthetic scheme of radiohalogenated α-MSH analogs

In this study, a neopentyl glycol (NpG) structure was used as the astatination scaffold because of its superior stability against in vivo deastatination compared to a representative astatination scaffold, the astatobenzoyl structure [15]. We assumed that the direct displacement of DO3A-monoamide with the NpG structure may be insufficient for designing ^211^At-labeled peptides. Thus, prior to studies using ^211^At, a ^125^I-labeled NpG group was conjugated to the N-terminus of GGNle-CycMSH_hex_ directly or via hydrophilic linkers to obtain four ^125^I-labeled GGNle-CycMSH_hex_ analogs (Fig. 1). D-Glu and D-Glu-D-Glu linkers were investigated because glutamate-based linkers have been used to prepare radiohalogenated peptides with high hydrophilicity [16–18]. However, the insertion of negative charges may impair the MC1R binding affinity of the parental peptide [19]. We investigated a D-Glu-D-Arg linker to neutralize the net charge by changing one of the D-Glus in the D-Glu-D-Glu linker to a positively charged D-Arg. A D-Glu-D-Arg linker was selected as the optimal linker by preliminary studies using ^125^I-labeled peptides. Finally, an ^211^At-labeled GGNle-CycMSH_hex_ analog with a D-Glu-D-Arg linker was prepared, and its potential as a TAT agent for metastatic melanoma was evaluated.

## Materials and methods

### General

The analytical methods for reversed-phase high-performance liquid chromatography (RP-HPLC) and reversed-phase thin-layer chromatography (RP-TLC), and the production procedure of ^211^At are described in the Supplementary information. The synthetic procedures of the precursors (**3** and N_3_-GGN**1**–N_3_-GGN**4**) for radiolabeling reactions and non-radioactive iodinated compounds (**5a** and NpG-GGN**1a**–NpG-GGN**4a**) for characterization of radiohalogenated compounds are also described in the Supplementary information. [^125^I]NaI (≃3.7 MBq/µL) was purchased from PerkinElmer.

### Radiosynthesis of [^125^I]5b

[^125^I]NaI (1.0 µL) was added to a solution of **3** in 1% *N*,*N*-diisopropylethylamine (DIEA)/acetonitrile (5 mM, 100 µL) and reacted for 1 h at 37 °C. After the reaction, a 4% aqueous solution of *p*-toluenesulfonic acid (TsOH, 100 µL) was added to the mixture, heated at 60 °C for 0.5 h, neutralized with 1 N NaOH aq, and purified by analytical RP-HPLC (system 1). The fractions containing the product were concentrated *in vacuo* to provide an aqueous solution of [^125^I]**5b**.

### Radiosynthesis of [^211^At]5c

A solution of ^211^At in acetonitrile (3–72 MBq/40 µL) and 10% sodium L-ascorbate (10 µL) was added successively to a solution of **3** in 2% DIEA/acetonitrile (10 mM 50 µL), and then reacted for 15 min at 37 °C. After the reaction, a 4% aqueous solution of TsOH (100 µL) was added to the mixture, and heated at 60 °C for 5 min. Purification was performed according to the procedure described for [^125^I]**5b**.

### Radiosyntheses of ^125^I/^211^At-labeled GGNle-CycMSH_hex_ analogs

Solutions of [^125^I]**5b** or [^211^At]**5c** in the mixture of acetonitrile and water (1/4, v/v), tris[(1-benzyl-1*H-*1,2,3-triazol-4-yl)methyl]amine (TBTA) in DMSO (15 mM), CuSO_4_ in water (6 mM), and sodium L-ascorbate in water (15 mM) were added successively to the solution of GGNle-CycMSH_hex_ analogs containing an azido group (1 mg/mL) in water with the volume ratio of 5/1/1/1/2 ([^125^I]**5b** or [^211^At]**5c**/TBTA/CuSO_4_/sodium L-ascorbate/GGNle-CycMSH_hex_ analogs). The mixture was stirred for 5 min at 40 °C and then purified by analytical RP-HPLC (system 2) to provide ^125^I/^211^At-labeled GGNle-CycMSH_hex_ analogs.

### Log D_7.4_ measurements

This experiment was performed according to a previously reported procedure with slight modifications [20]. In brief, an aliquot of radiolabeled GGNle-CycMSH_hex_ analogs (50 kBq/5 µL) was mixed with equal amounts (1.5 mL) of 1-octanol and 25 mM phosphate buffer (pH 7.4). The mixture was vortexed for 1 min and then allowed to stand for 1 min. After repeating the procedure five times, the mixture was centrifuged at 1,500 *g* for 10 min. Aliquots of 0.5 mL were taken from each phase and their radioactivity was measured with an auto-well γ counter (Wizard 3, PerkinElmer). The partition coefficient was determined by calculating the ratio of counts per minute in the 1-octanol phase to that in the buffer phase and expressed as a common logarithm. The results represent the mean ± SD for each compound.

### In vitro stability in murine plasma

A solution of radioiodinated GGNle-CycMSH_hex_ analogs (30 kBq/10 µL) or [^211^At]NpG-GGN**4c** (100 kBq/10 µL) was added to 90 µL of freshly prepared murine plasma. After incubation for 24 h at 37 °C, acetonitrile (100 µL) was added to the solution, and the mixture was centrifuged (10,000 *g*, 5 min). The supernatant was analyzed by RP-HPLC (system 3, *n* = 3).

### Receptor binding assays

B16F10 cells were obtained from the Cell Resource Center for Biomedical Research (Institute of Development, Aging and Cancer Tohoku University). B16F10 cells were cultivated in RPMI 1640 medium (Nacalai Tesque) supplemented with 10% fetal bovine serum (Thermo Scientific) in a humidified atmosphere containing 5% carbon dioxide at 37 °C. B16F10 cells (5 × 10^5^ cells/well) were seeded in a 24-well poly-D-lysine coated plate (Corning) overnight. After the growth medium was removed, a reaction buffer containing 4.8 mg/mL HEPES, 100 U/mL penicillin, 1 mg/mL streptomycin and 2 mg/mL bovine serum albumin was added. Approximately 30,000 counts per minute of [^125^I]I-Tyr^2^-NDP-MSH and 0.5 pM to 5 µM of the peptides (NpG-GGN**1a**–NpG-GGN**4a** and In-DOTA-GGNle-CycMSH_hex_**)** were added to each well. The reaction mixture was incubated at 37 °C for 1 h. After incubation, the binding medium was removed, and the cells were rinsed twice with ice-cold phosphate-buffered saline (PBS). The cells were harvested using 0.25% trypsin solution, and the radioactivity was determined using an auto-well gamma counter. Ki values were calculated using GraphPad Prism 8.4.3 (GraphPad Software).

### Biodistribution in tumor-bearing mice

The animal experiments were conducted according to a protocol reviewed and approved by the Chiba University Animal Care Committee (Permit No. 4–183). Five-week-old C57BL/6 mice were subcutaneously xenografted in their right hind legs by injecting B16F10 cells (1.0 × 10^6^ cells) suspended in Matrigel (BD Biosciences). When the tumor diameter achieved approximately 10 mm, the mice were subjected to biodistribution studies. Groups of four mice were sacrificed at each postinjection time point. A solution of each radiohalogenated GGNle-CycMSH_hex_ analog or [^111^In]In-DOTA-GGNle-CycMSH_hex_ (7.4 kBq/100 µL) was injected into the tumor-bearing mice via the tail vein, and the mice were sacrificed at 3 h postinjection. A solution of [^125^I]NpG-GGN**4b** (7.4 kBq) or [^211^At]NpG-GGN**4c** (40 or 100 kBq) was also injected, and the mice were sacrificed at 1, 3, and 15 h postinjection. Injected radioactivity of [^211^At]NpG-GGN**4c** was 40 kBq for 1 and 3 h postinjection groups and 100 kBq for 24 postinjection group. Blocking studies were performed by co-injection of NDP-MSH (10 µg per mouse) simultaneously with [^125^I]NpG-GGN**4b** (7.4 kBq) or [^211^At]NpG-GGN**4c** (40 kBq), and the mice were sacrificed at 3 h postinjection. The organs of interest were removed, weighed, and radioactivity was estimated using an auto-well gamma counter.

### Urine analysis

A solution of [^125^I]NpG-GGN**4b** (200 kBq/100 µL) or [^211^At]NpG-GGN**4c** (400 kBq/100 µL) was injected intravenously into six-week-old male C57BL/6 mice, and the urine samples were collected for 3 h. Acetonitrile (100 µL) was added to the urine sample (100 µL), and the mixture was centrifuged (10,000 *g*, 5 min). The supernatant was analyzed by RP-HPLC (system 3).

### Therapeutic study

The animal experiments were conducted according to a protocol reviewed and approved by the Chiba University Animal Care Committee (Permit No. 5–225). B16F10 cells were implanted into five-week-old C57BL/6 mice in the same manner as described in the biodistribution study. When the tumor volume achieved approximately 150–200 mm^3^, the mice were subjected to therapeutic studies. [^211^At]NpG-GGN**4c** (0.4 or 1.0 MBq/100 µL) or saline (100 µL) was injected into the tumor-bearing mice via the tail vein (*n* = 6). Tumor size and body weights were measured six days per week after the injection. Tumor volume (mm^3^) was calculated as 4π/3×(width/2)^2^×(length/2). In cases of tumor volumes greater than 1,500 mm^3^, the mice were euthanized humanely according to our institutional guidelines. The survival curve was estimated for each group using the Kaplan–Meier method.

### Statistical analysis

Statistical analyses were performed using GraphPad Prism 8.4.3. Data are expressed as the means ± SD where appropriate. Results were statistically analyzed using Student’s t-test or Tukey’s multiple comparison test. Efficacy data were analyzed using Kaplan–Meier survival curves with a log-rank test. Statistical significance was set at *p* < 0.05.

## Results

### Radiolabeling

Precursor **3** was prepared from pentaerythritol in three steps, with an overall yield of 6.3% (Supplementary Scheme 1). ^211^At was produced by the ^209^Bi(α, 2n)^211^At reaction and isolated from the irradiated target using the dry distillation method. [^211^At]**5c** was prepared via a one-pot, two-step reaction (Fig. 1). Four CycMSH_hex_ analogs containing an azide group (N_3_-GGN**1**–N_3_-GGN**4**) were synthesized using conventional Fmoc solid-phase peptide synthesis. [^125^I]**5b** and [^211^At]**5c** were used for the copper-catalyzed azide-alkyne cycloaddition (CuAAC) reactions with azido peptides to produce radiohalogenated α-MSH analogs ([^125^I]NpG-GGN**1b**–[^125^I]NpG-GGN**4b** and [^211^At]NpG-GGN**4c**). The CuAAC reaction was performed at 40 °C for 5 min, and the radiochemical conversion (RCC) and radiochemical yield (RCY) are listed in Table 1. Because the expression level of MC1R on melanoma cells is low, radiolabeled α-MSH analogs require HPLC purification for isolation from non-labeled peptides [13]. We also performed HPLC purification of [^111^In]In-DOTA-GGNle-CycMSH_hex_ and determined RCC and RCY (Table 1). All radiohalogenated compounds developed in this study were characterized by the corresponding non-radioactive iodinated compounds (**5a** and NpG-GGN**1a**–NpG-GGN**4a**) (Supplementary Figs. 1–7).

**Table 1:** Radiochemistry data of the CuAAC reagents and radiolabeled α-MSH analogs^*a*^

Compounds	RCCs (%)^*b*^	RCYs (%)^*c*^	Log D_7.4_	Plasma stability (%)^*d*^
[^125^I]**5b**	78.1 ± 5.5^*e, g*^	40.4 ± 3.4^*f, g*^	N.D.	N.D.
[^211^At]**5c**	95.1 ± 2.9^*e, g*^	66.4 ± 9.7^*f, g*^	N.D.	N.D.
[^125^I]NpG-GGN**1b**	88.3 ± 3.5	44.8 ± 4.9	−0.72 ± 0.02	71.7 ± 1.8
[^125^I]NpG-GGN**2b**	86.7 ± 5.2	40.3 ± 4.8	−0.91 ± 0.01	95.2 ± 1.6
[^125^I]NpG-GGN**3b**	90.8 ± 2.4	49.5 ± 2.6	−2.31 ± 0.01	96.1 ± 0.4
[^125^I]NpG-GGN**4b**	89.0 ± 4.6^*g*^	44.4 ± 3.6^*g*^	−2.19 ± 0.02	96.9 ± 0.8
[^211^At]NpG-GGN**4c**	88.3 ± 3.5^*g*^	43.0 ± 3.4^*g*^	−2.03 ± 0.11	95.6 ± 0.2
[^111^In]In-DOTA-GGNle-CycMSH_hex_	96.8 ± 1.0	68.8 ± 2.1	−3.45 ± 0.17	N.D.

^*a*^Data represent the mean ± SD (*n* = 3 with some exceptions^*g*^)

^*b*^Determined by RP-HPLC

^*c*^Shown as the decay-corrected values

^*d*^Determined by in vitro experiments for 24 h

^*e*^Determined as the overall RCC of two steps reaction

^*f*^Calculated as the overall RCY of two steps reaction

^*g*^Data represent the mean ± SD (*n* = 5)

### Log D_7.4_ measurements

The Log D_7.4_ values are listed in Table 1. Correlating with the number of glutamate molecules, the log D_7.4_ value was reduced. The introduction of one glutamate molecule ([^125^I]NpG-GGN**2b**) did not considerably enhance the hydrophilicity of [^125^I]NpG-GGN**1b** (−0.91 ± 0.01 vs. −0.72 ± 0.02); however, the addition of two glutamate molecules ([^125^I]NpG-GGN**3b**) did (−2.31 ± 0.01). The log D_7.4_ value of [^125^I]NpG-GGN**4b** (−2.19 ± 0.02) was similar to that of [^125^I]NpG-GGN**3b** (−2.31 ± 0.01). [^211^At]NpG-GGN**4c** showed similar but slightly higher log D_7.4_ values than that of [^125^I]NpG-GGN**4b**.

### In vitro stability in murine plasma

After incubation in freshly prepared murine plasma for 24 h, radiohalogenated GGNle-CycMSH_hex_ analogs except [^125^I]NpG-GGN**1b** remained intact as determined by RP-HPLC (> 95%), and 71.7 ± 1.8% of radioactivity remained intact for [^125^I]NpG-GGN**1b** (Table 1 and supplementary Fig. 9).

### In vitro competitive inhibition assays

**Fig. 2:**
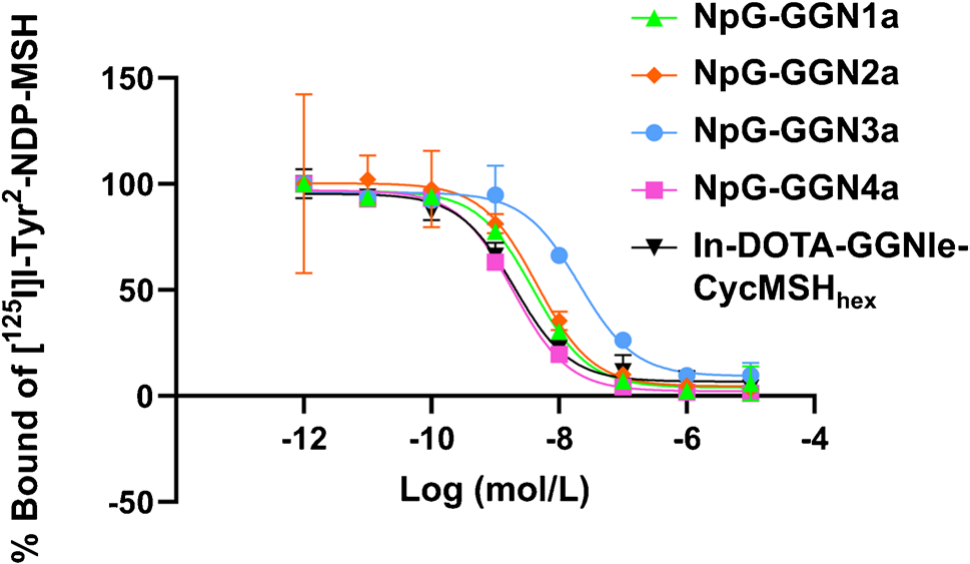
In vitro competitive inhibition assays of [^125^I]I-Tyr^2^-NDP-MSH bound to B16F10 melanoma cells by α-MSH analogs. The Ki values (95% confidential intervals) of NpG-GGN**1a**, NpG-GGN**2a**, NpG-GGN**3a**, NpG-GGN**4a**, and In-DOTA-GGNle-CycMSH_hex_ were 2.81 (2.26–3.47), 3.24 (1.31–7.65), 14.9 (10.4–21.6), 1.37 (1.17–1.62), and 1.44 (1.03–2.05) nM, respectively

The binding affinities of the NpG-conjugated α-MSH analogs to B16F10-melanoma cells were evaluated using competition binding assays with [^125^I]I-Tyr^2^-NDP-MSH and compared with those of In-DOTA-GGNle-CycMSH_hex_. The Ki value of NpG-GGN**3a** (14.9 nM) was 5.3 and 10 times higher than that of NpG-GGN**1a** (2.81 nM) and In-DOTA-GGNle-CycMSH_hex_ (1.44 nM), respectively (Fig. 2). The Ki value of NpG-GGN**4a** (1.37 nM) was comparable with that of In-DOTA-GGNle-CycMSH_hex_ (Fig. 2).

### Biodistribution of ^125^I-labeled α-MSH analogs in the tumor-bearing mice

**Fig. 3:**
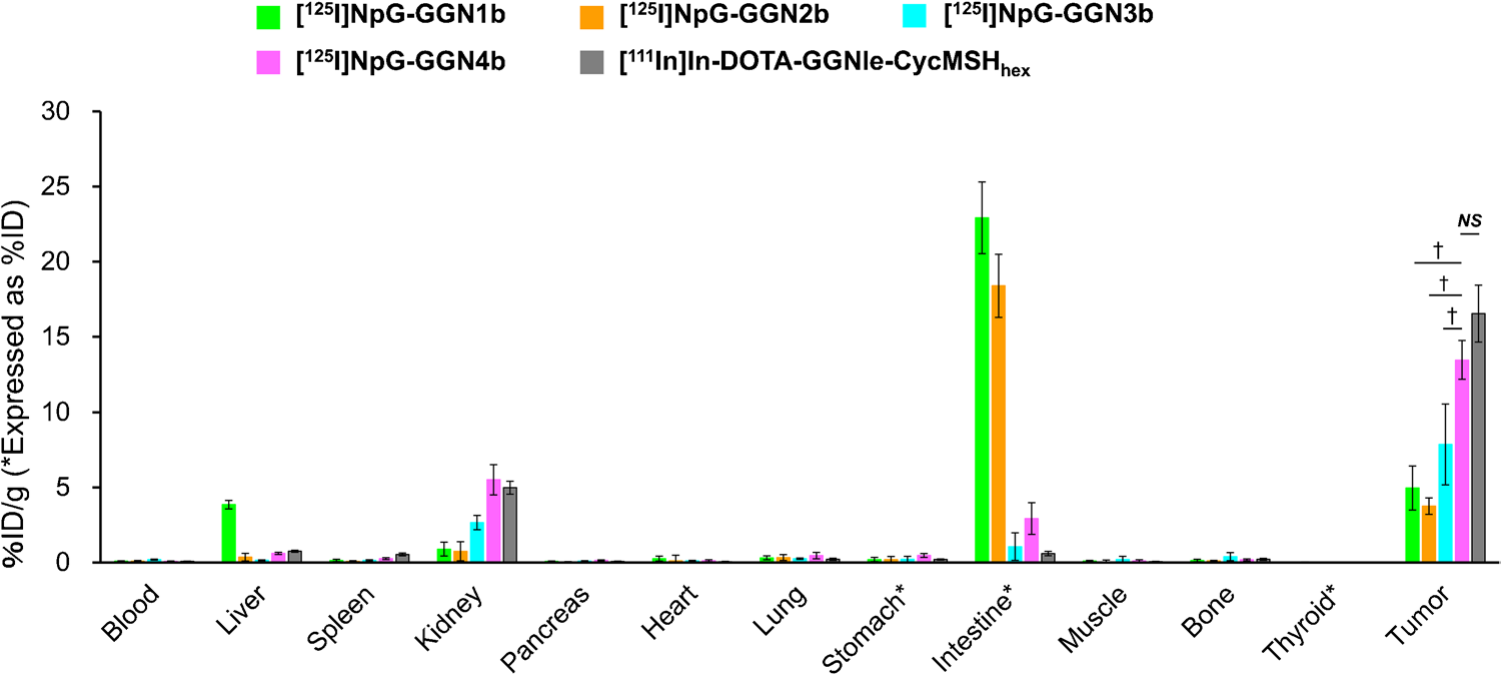
Biodistribution of radioactivity at 3 h after injection of ^125^I- and ^111^In-labeled α-MSH analogs to B16F10 tumor-bearing mice. Statistical significances in the tumor accumulations were shown in the figure; *p* < 0.05 compared with [^125^I]NpG-GGN**4b** (^†^). No significant difference was observed between the tumor accumulation of [^125^I]NpG-GGN**4b** and [^111^In]In-DOTA-GGNle-CycMSH_hex_, which is also shown in the figure (NS). Other significances are listed in Supplementary Table 1

The localization of radioactivity in the organs after injecting the four ^125^I-labeled and ^111^In-labeled α-MSH analogs is shown in Fig. 3, and the detailed results are shown in Supplementary Table 1. [^125^I]NpG-GGN**1b** was eliminated via the hepatobiliary route, resulting in high accumulation in the liver and intestine. [^125^I]NpG-GGN**2b** exhibited lower accumulation in the liver than that in [^125^I]NpG-GGN**1b** but comparable accumulation in the intestine. [^125^I]NpG-GGN**3b** and [^125^I]NpG-GGN**4b** dramatically reduced radioactivity levels in the liver and intestine compared to [^125^I]NpG-GGN**1b**. Consistent with the MC1R affinity determined in an in vitro study, [^125^I]NpG-GGN**4b** exhibited significantly higher accumulation in the tumor than [^125^I]NpG-GGN**3b** (*p* < 0.05) and was comparable to [^111^In]In-DOTA-GGNle-CycMSH_hex_.

### Comparative biodistribution of [^211^At]NpG-GGN4c and [^125^I]NpG-GGN4b in the tumor-bearing mice

**Fig. 4:**
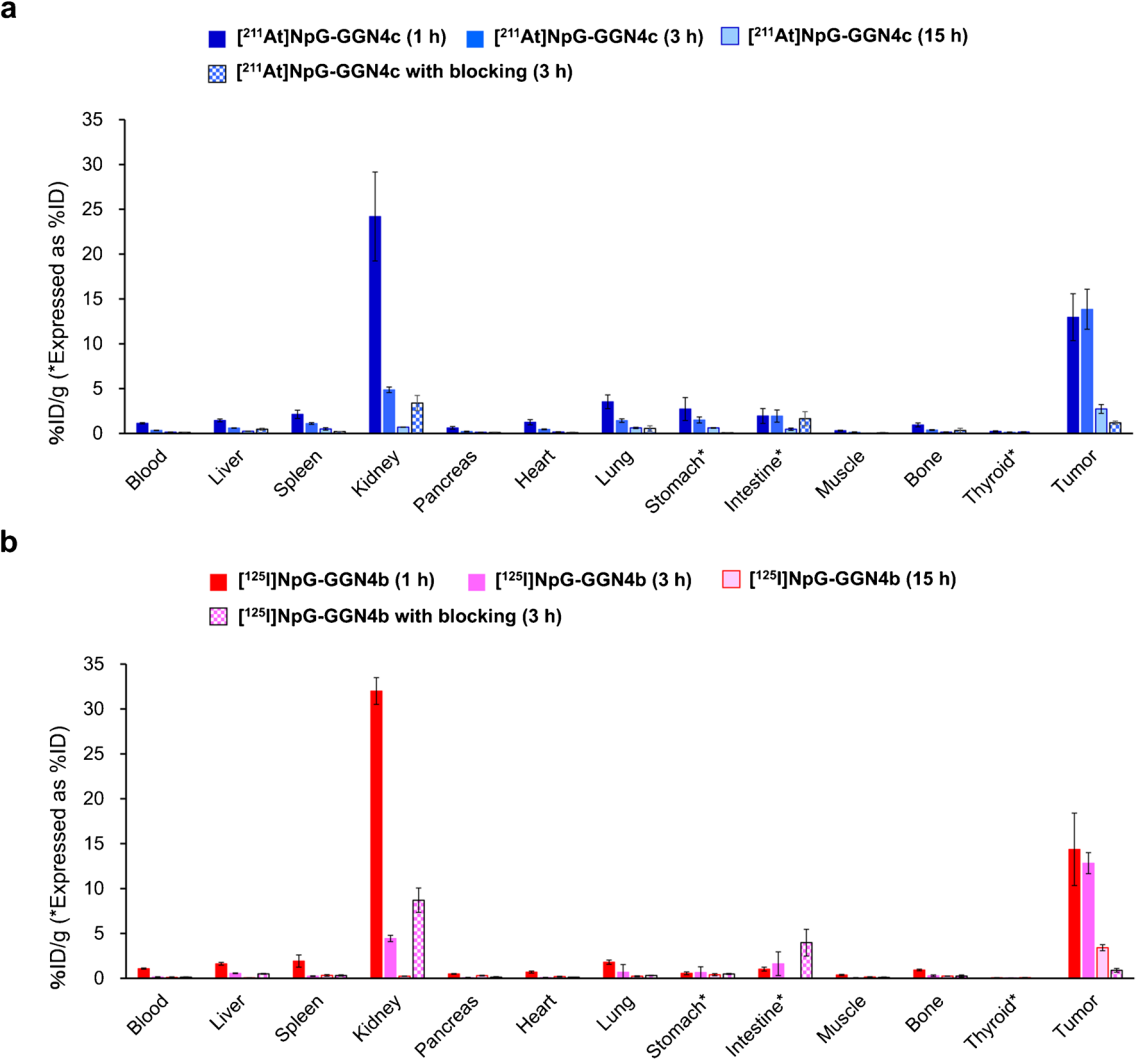
Biodistribution of radioactivity at 1, 3, and 15 h after injection of (a) [^211^At]NpG-GGN**4c** and (b) [^125^I]NpG-GGN**4b** to B16F10-tumor-bearing mice. Blocking study was performed at 3 h postinjection. Significant differences are listed in Supplementary Table 2

The localization of radioactivity in the organs after injecting [^211^At]NpG-GGN**4c** and [^125^I]NpG-GGN**4b** is shown in Fig. 4, and detailed results are shown in Supplementary Table 2. Although [^211^At]NpG-GGN**4c** showed high radioactivity levels in the kidney at 1 h postinjection, these levels were rapidly reduced by 80% from 1 to 3 h postinjection. Radioactivity levels in the tumors were maintaned from 1 to 3 h postinjection, but tumor accumulation was then reduced by 80% from 3 to 15 h postinjection. Radioactivity levels of [^211^At]NpG-GGN**4c** in the stomach and thyroid were significantly higher than those of [^125^I]NpG-GGN**4b**. Tumor accumulation was significantly inhibited by co-injection of excess amounts of NDP-MSH (*p* < 0.05).

### Urine analyses of [^211^At]NpG-GGN4c and [^125^I]NpG-GGN4b

The urine samples were prepared after the precipitation of proteins, and over 90% of radioactivity was collected in the supernatant. In each RP-HPLC analysis of the urine samples of [^211^At]NpG-GGN**4c** and [^125^I]NpG-GGN**4b**, intact peptide was observed as the main peak (Supplementary Fig. 10).

### Therapeutic study of [^211^At]NpG-GGN4c

**Fig. 5:**
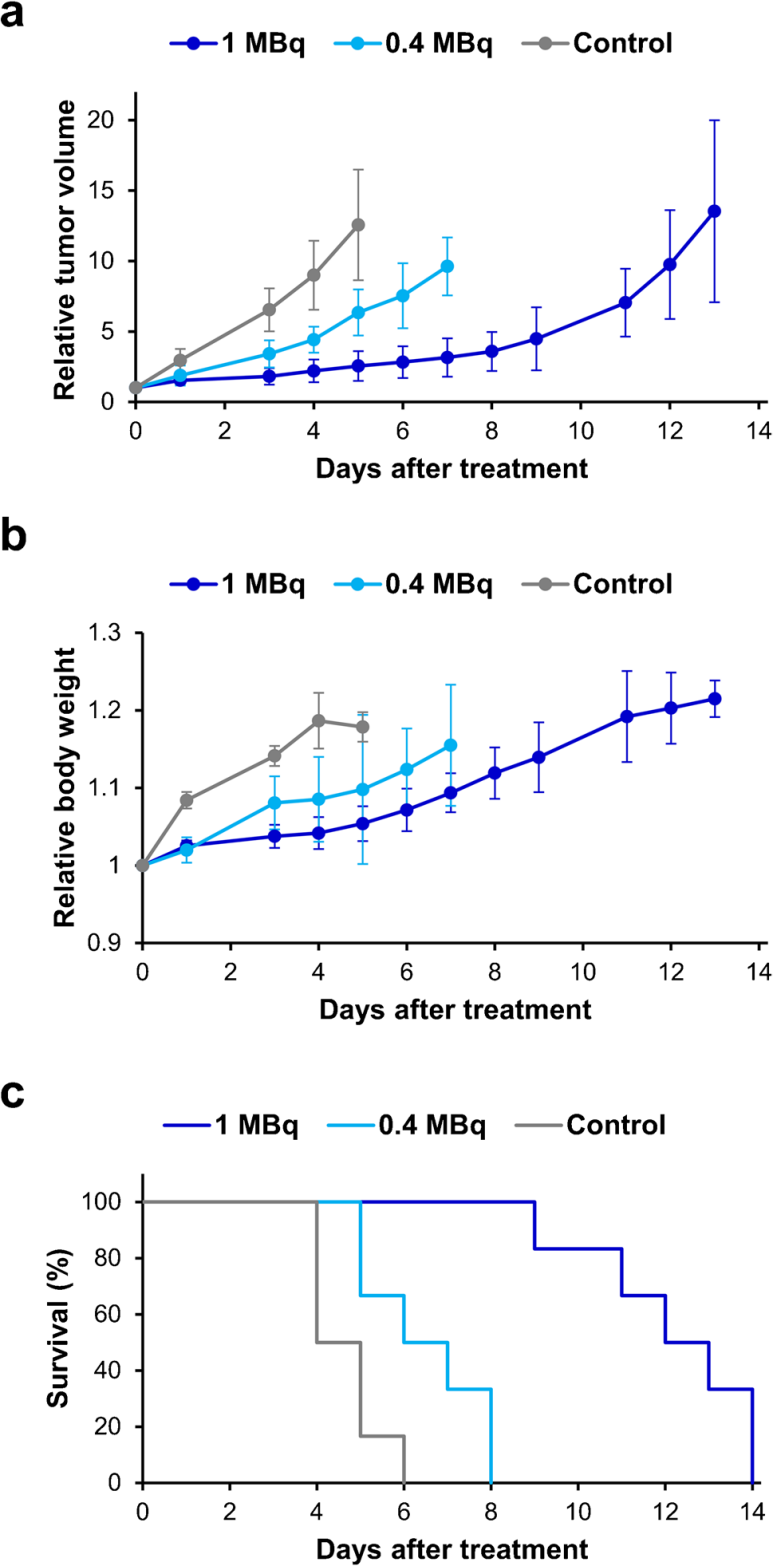
Therapeutic effect of [^211^At]NpG-GGN**4c** in B16F10 tumor-bearing mice. **a** Tumor volume, **b** body weight, and **c** Kaplan–Meier survival curves after injection of [^211^At]NpG-GGN**4c** (1 or 0.4 MBq, *n* = 6) or saline (control, *n* = 6)

[^211^At]NpG-GGN**4c** inhibited tumor growth in a dose-dependent manner, and both injection doses showed significant inhibition compared to the control group on all days after treatment (excluding day 0) (*p* < 0.05) (Fig. 5a). Body weight loss was not observed even in the group that received 1 MBq of [^211^At]NpG-GGN**4c** (Fig. 5b). Kaplan–Meier survival analysis showed that [^211^At]NpG-GGN**4c** treatment significantly improved the survival of mice compared to the control group (*p* < 0.05) (Fig. 5c).

## Discussion

In our previous study to develop an ^211^At-labeled agent targeting prostate-specific membrane antigen (PSMA), we prepared a precursor whose five carboxylic acids were protected by *tert*-butyl groups [18]. If we prepare the ^211^At-labeled α-MSH analog by a similar synthetic procedure, we must prepare the precursor of α-MSH analog whose reactive functional groups are all protected. However, its molecular weight is approximately twice that of the PSMA precursor, and its synthesis is challenging. Thus, we prepared CuAAC reagents of ^125^I ([^125^I]**5b**) and ^211^At ([^211^At]**5c**), and subsequently conjugated them with α-MSH analog by the CuAAC reaction to produce ^125^I/^211^At-labeled α-MSH analogs. This approach is helpful to efficiently screen hydrophilic linkers.

It is well known that several chemical species of ^211^At exist in a solution of ^211^At extracted from the dry distillation [21]. Because the addition of ascorbic acid is effective in reducing oxidized species to [^211^At]At^−^ [22], sodium ascorbate was added to the astatination reaction. [^211^At]**5c** was prepared via a one-pot, two-step reaction, considering its short half-life (Fig. 1). Although the reaction time required to synthesize [^211^At]**4c** (15 min) was shorter than that of [^125^I]**4b** (60 min), [^211^At]**5c** was obtained in higher radiochemical yields (RCYs) than [^125^I]**5b** (Table 1). These results are consistent with previous studies [15, 23] and the theory that the heavier halogen is more reactive than the lighter one in a solution containing water. While [^211^At]At^−^ is used for the astatination reaction of NpG derivatives, [^211^At]At^+^ is used for the reaction to prepare the astatobenzoyl compounds. The reaction using [^211^At]At^−^ is reported to have the advantage in reproducibility and efficiency [24]. Although the direct comparison was not investigated in this study, [^211^At]**5c** was given in high RCCs, and such an advantage might also apply to the reaction of NpG derivatives. The high RCCs of the CuAAC reaction were confirmed using RP-HPLC analysis; however, the RCYs were moderate (Table 1). The differences between RCCs and RCYs were also observed for [^111^In]In-DOTA-GGNle-CycMSH_hex_ prepared in this study. In addition, moderate RCYs of ^68^Ga-labeled α-MSH analogs were reported by another group [25]. We also determined RCCs using RP-TLC (Supplementary Fig. 8), which almost corresponded to the results obtained from RP-HPLC. These results imply that after the CuAAC reaction, the samples did not contain any unidentified chemical species that were not eluted from the RP-HPLC column. When the isolated [^125^I]NpG-GGN**4b** was reinjected to RP-HPLC, the collected radioactivity was impaired (recovery yields: 66.3 ± 1.7%, *n* = 3). These results suggest that radiolabeled α-MSH analogs tend to stack in the HPLC column or be absorbed in the tube, which originates from the physicochemical properties of the parental α-MSH analog. Because this step proceeds at high RCCs, optimizing the purification method may improve the RCYs.

The log D_7.4_ values of [^125^I]NpG-GGN**1b** and [^125^I]NpG-GGN**2b** were comparable to those of α-MSH analogs eliminated via the hepatobiliary route [13, 14]. The high accumulation in the intestine correlated with their log D_7.4_ values. The log D_7.4_ values of [^125^I]NpG-GGN**3b** (−2.31 ± 0.01) and [^125^I]NpG-GGN**4b** (−2.19 ± 0.02) were higher than that of [^111^In]In-DOTA-GGNle-CycMSH_hex_ (−3.45 ± 0.17), but comparable to that of the ^64^Cu-labeled α-MSH analog (−2.30 ± 0.00) which was rapidly excreted into the urine [26]. Indeed, [^125^I]NpG-GGN**3b** and [^125^I]NpG-GGN**4b** showed low accumulation in the liver and intestine. Although [^125^I]NpG-GGN**1b** showed a small degree of degradation after in vitro plasma stability experiments, the other ^125^I-labeled GGNle-CycMSH_hex_ analogs showed high stability (Table 1). The results suggest that insertion of D-amino acid(s) would enhance stability of ^125^I-labeled GGNle-CycMSH_hex_ analogs.

Displacing the Gly-Gly-Nle of DOTA-GGNle-CycMSH_hex_ with a sequence containing Glu reduces its binding affinity for MC1R probably due to the electrostatic interaction between Glu and Arg in CycMSH_hex_ [19]. The IC_50_ value of DOTA-GENle-CycMSH_hex_ that used the Gly-Glu-Nle linker was 5.5 times that of DOTA-GGNle-CycMSH_hex_ [19]. In this study, linkers containing D-Glu were conjugated to the N-terminus of GGNle-CycMSH_hex_ to keep a distance from CycMSH_hex_. As a result, NpG-GGN**2a** showed the binding affinity comparable to that of NpG-GGN**1a**. However, NpG-GGN**3a** impaired the binding affinity of the parental peptide. The two additional negative charges of NpG-GGN**3a** might enhance the electrostatic interaction with Arg in CycMSH_hex_. In contrast, inserting a positively charged Arg increase the binding affinity of the parental peptide, and the Ki value of NpG-GGN**4a** was 2.1 times lower than that of NpG-GGN**1a**. In the biodistribution study, [^125^I]NpG-GGN**3b** showed the second-highest tumor accumulation among the four radioiodinated α-MSH analogs despite having the lowest affinity for MC1R (Fig. 3a). Hence, biodistribution profiles that do not impair tumor accumulation by avoiding rapid elimination via the hepatobiliary route, as well as high tumor affinity, are important for the development of radiolabeled α-MSH analogs. These results suggest that inserting a peptide linker consisting of at least two hydrophilic amino acids is suitable for preparing GGNle-CycMSH_hex_ analogs that are radiohalogenated via an NpG scaffold.

Thus, [^211^At]NpG-GGN**4c** was prepared and used for further evaluation. The high accumulation of [^211^At]NpG-GGN**4c** in the tumor was significantly reduced by MC1R inhibition (*p* < 0.05) (Fig. 3). The results indicated that the high affinity of [^211^At]NpG-GGN**4c** contributed to high MC1R-specific accumulation in the tumor. Although tumor accumulation decreased from 3 to 15 h, tumor retention at early time points could be valuabled for TAT using ^211^At-labeled agents because of the short half-life of ^211^At. [^211^At]NpG-GGN**4c** showed biodistribution profiles similar to that of [^125^I]NpG-GGN**4b**; however, significant differences in radioactivity accumulation were observed in several organs, such as the thyroid and stomach (*p* < 0.05) (Supplementary Table 2). In general, ^211^At-labeled compounds with low stability against in vivo deastatination produce free [^211^At]astatide rapidly after injection, resulting in increased radioactivity in the thyroid and stomach [15, 27, 28]. However, accumulation of [^211^At]NpG-GGN**4c** in these tissues was inhibited in the blocking study (Supplementary Table 2). Notably, [^211^At]NpG-GGN**4c** exhibited high stability against in vivo deastatination, at least in the blocking studies. If [^211^At]NpG-GGN**4c** produces free [^211^At]astatide in the tumor, accumulation of radioactivity in the tumor is likely to be impaired. However, no significant difference was observed in the tumor accumulation of [^211^At]NpG-GGN**4c** and [^125^I]NpG-GGN**4b** at any of postinjection time points investigated in this study (Fig. 4). [^211^At]NpG-GGN**4c** showed higher radioactivity levels in the thyroid than those of [^125^I]NpG-GGN**4b**, but the levels were still very low. Whereas, accumulation of radioactivity in the stomach was moderate. Although no significant difference was observed in the blocking study of [^125^I]NpG-GGN**4b**, ^90^Y- and ^203^Pb-labeled DOTA-GGNle-CycMSH_hex_ reduced radioactivity levels in the stomach by blocking MC1R in B16F10 tumor-bearing mice [29, 30]. These results suggest that MC1R-specific accumulation may be involved in the accumulation of GGNle-CycMSH_hex_ analogs in the stomach. Therefore, accumulation in the stomach may be unsuitable as an index of in vivo deastatination of [^211^At]NpG-GGN**4c**. In this study, the other index, radioactivity levels in the thyroid were very low, suggesting that [^211^At]NpG-GGN**4c** would be sufficiently stable and acceptable for therapeutic applications. In addition, the intact [^211^At]NpG-GGN**4c** was observed as the main peak in the urine analysis, supporting sufficient stability of [^211^At]NpG-GGN**4c** (Supplementary Fig. 10).

The tumor size used in this study (178 ± 60 mm^3^) was larger than that typically used in therapuetic studies of B16F10 tumor-bearing mice (~ 60 mm^3^) [31, 32]. Inoculated B16F10 tumors in the control group grew rapidly in C57BL/6 mice, reaching 1,500 mm^3^ within six days of saline administration (Fig. 5a). Rapid tumor proliferation and relatively large tumor size were a concern in limiting the therapeutic effects. However, [^211^At]NpG-GGN**4c** significantly inhibited tumor growth, indicating high therapeutic efficiency of [^211^At]NpG-GGN**4c** (Fig. 5a). Temporary body weight loss was observed in several preclinical studies with ^211^At-labeled agents [31, 33] but not observed in this study (Fig. 5b). [^211^At]NpG-GGN**4c** showed high renal accumulation, although it was rapidly eliminated. Otherwise, mild accumulation of radioactivity was observed in the stomach. Recently, the toxicity of an ^211^At-labeled agent targeting PSMA, [^211^At]PSMA-5, has been thoroughly investigated [34, 35]. It caused mild in vivo deastatination and showed remarkably higher and more persistent radioactivity in the kidney than [^211^At]NpG-GGN**4c** did. However, no serious toxicity was observed even when 1 MBq of [^211^At]PSMA-5 was administrated [34]. Although detailed analysis of the side effects including histopathological evaluation is needed before clinical application, these findings suggest that the toxicity of [^211^At]NpG-GGN**4c** may be tolerable.

## Conclusion

In this study, we developed a novel ^211^At-labeled peptide targeting MC1R, [^211^At]NpG-GGN**4c**. It exhibited high tumor accumulation, rapid clearance from the kidney, and low radioactivity levels in other off-target tissues. [^211^At]NpG-GGN**4c** also demonstrated a dose-dependent therapeutic effect in B16F10 tumor-bearing mice without apparent body weight loss. These results suggest that [^211^At]NpG-GGN**4c** is a promising TAT agent for the treatment of metastatic melanoma.

## Supplementary information

Below is the link to the electronic supplementary material.

ESM 1 (DOCX 8.21 MB)

## Data Availability

Data and materials are available from the corresponding authors upon reasonable request.
