# Supplementary material for: An 211At-labeled alpha-melanocyte stimulating hormone peptide analog for targeted alpha therapy of metastatic melanoma
Source: Eur J Nucl Med Mol Imaging. 2025 Jan 20;52(6):2107–17. doi: 10.1007/s00259-024-07056-3 (PMC12014842; doi:10.1007/s00259-024-07056-3)

Supplementary Information

**An ^211^At-labeled alpha-melanocyte stimulating hormone peptide analog for targeted alpha therapy of metastatic melanoma**

Hiroyuki Suzuki,*^1^ Saki Yamashita,^1^ Shoko Tanaka,^1^ Kento Kannaka,^1^ Ichiro Sasaki,^2^ Yasuhiro Ohshima,^2^ Shigeki Watanabe,^2^ Kazuhiro Ooe,^3^ Tadashi Watabe,^3,4^ Noriko S. Ishioka,^2^ Hiroshi Tanaka^5,6^ and Tomoya Uehara^1^

^1^Graduate School of Pharmaceutical Sciences, Chiba University, 1-8-1 Inohana, Chuo-ku, Chiba 260-8675, Japan

^2^Department of Quantum-Applied Biosciences, Takasaki Institute for Advanced Quantum Science, National Institutes for Quantum Science and Technology, 1233 Watanuki, Takasaki, Gunma 370-1292, Japan

^3^Institute for Radiation Sciences, Osaka University, 1-1 Machikaneyama, Toyonaka, Osaka 560-0043, Japan

^4^Department of Radiology, Graduate School of Medicine, Osaka University, 2-2 Yamadaoka, Suita, Osaka 565-0871, Japan

^5^Faculty of Pharmacy, Juntendo University, 6-8-1 Hinode, Urayasu, Chiba, 279-0013 Japan

^6^Department of Chemical Science and Engineering, Institute of Science Tokyo, 2-12-1 Ookayama, Meguro-ku, Tokyo 152-8552, Japan

**Corresponding author**

*Hiroyuki Suzuki

Graduate School of Pharmaceutical Sciences, Chiba University,

1-8-1 Inohana, Chuo-ku, Chiba 260-8675, Japan.

Phone: +81-43-226-2898

Fax: +81-43-226-2898

E-mail: h.suzuki@chiba-u.jp

**Table of Contents**

[General 3](#_Toc184827054)

[Production of ^211^At 3](#_Toc184827055)

[Syntheses 4](#_Toc184827056)

[**(2,2-Dimethyl-1,3-dioxane-5,5-diyl)dimethanol (1) 4**](#_Toc184827057)

[**(2,2-dimethyl-5-((prop-2-yn-1-yloxy)methyl)-1,3-dioxan-5-yl)methanol (2) 4**](#_Toc184827058)

[**(2,2-dimethyl-5-((prop-2-yn-1-yloxy)methyl)-1,3-dioxan-5-yl)methyl trifluoromethanesulfonate (3) 5**](#_Toc184827059)

[**5-(iodomethyl)-2,2-dimethyl-5-((prop-2-yn-1-yloxy)methyl)-1,3-dioxane (4a) 5**](#_Toc184827060)

[**2-(iodomethyl)-2-((prop-2-yn-1-yloxy)methyl)propane-1,3-diol (5a) 5**](#_Toc184827061)

[**GGNle-CycMSH_hex_ analogs including an azido group 6**](#_Toc184827062)

[**Non-radioactive iodinated GGNle-CycMSH_hex_ analogs 6**](#_Toc184827063)

[References 7](#_Toc184827064)

[Supplementary Tables 8](#_Toc184827065)

[Supplementary Figures 11](#_Toc184827066)

[**Characterization and purity check for radiohalogenated compounds 11**](#_Toc184827067)

[**[^125^I]5b 11**](#_Toc184827068)

[**[^211^At]5c 12**](#_Toc184827069)

[**[^125^I]NpG-GGN1b 13**](#_Toc184827070)

[**[^125^I]NpG-GGN2b 14**](#_Toc184827071)

[**[^125^I]NpG-GGN3b 15**](#_Toc184827072)

[**[^125^I]NpG-GGN4b 16**](#_Toc184827073)

[**[^211^At]NpG-GGN4c 17**](#_Toc184827074)

[**RP-TLC radiochromatograms for analyzing CuAAC reaction 18**](#_Toc184827075)

[***In vitro* stability in murine plasma 19**](#_Toc184827076)

[**Urine analysis 21**](#_Toc184827077)

[**Therapeutic effect of [^211^At]NpG-GGN4c in individual mice 22**](#_Toc184827078)

[MS spectra 23](#_Toc184827079)

[NMR Spectra 24](#_Toc184827080)

# **General**

^1^H-NMR spectra were recorded on a JEOL JNM-ECS 400 spectrometer (JEOL, Tokyo, Japan). ^13^C-NMR spectra were obtained by a JEOL ECS-600 spectrometer (JEOL). Mass spectrometry was performed using an AccuTOF LC-plus (JMS-T100LP, JEOL). The spots on TLC plates were visualized by UV at 254 nm or phosphomolybdic acid staining. [^125^I]NaI (≃3.7 MBq/μL) was purchased from Perkin Elmer (Waltham, MA, USA). Analytical reversed-phase (RP)-HPLC (L-7100 pump, Hitachi, Tokyo, Japan) was performed with a Unison US-C18 column (4.6 × 150 mm, Imtakt, Kyoto, Japan) at flow rate of 1.0 mL/min with a linear gradient starting from 80% A (water) and 20% B (acetonitrile) to 50% B at 25 min, and then to 100% B at 30 min (system 1), or from 80% C (0.1% aqueous trifluoroacetic acid (TFA)) and 20% D (acetonitrile with 0.1% TFA) to 25% D at 5 min to 35% D at 35 min, and then to 100% D at 40 min (system 2), or with an Asahipak C8P-50 4D column (4.6 × 150 mm, Resonac, Tokyo, Japan) from 90% C and 10% D to 50% D at 16 min to 100% D at 18 min, and then to 100% D at 20 min (system 3). The effluent was monitored by detection at 254 nm with a UV detector (L-7405, Hitachi, Tokyo, Japan) coupled to a NaI(Tl) radioactivity detector (Gabi star, Raytest, Strubenhardt, Germany). In the *in vitro* plasma stability studies, the effluent was collected with a fraction collector (Frac-920, GE Healthcare Japan, Tokyo) at 1.0 min intervals after monitoring by NaI(Tl) radioactivity detector, and the radioactivity counts in each fraction were determined with an automated gamma well counter (Wizard 3, PerkinElmer Japan, Yokohama, Japan). Preparative RP-HPLC was performed on a Cadenza 5CD-C18 column (20 × 150 mm, Imtakt) at a flow rate of 5.0 mL/min with a linear gradient starting from 80% C and 20% D to 40% B at 30 min, and then to 100% B at 40 min (system 4), or from 25% B to 35% B at 30 min, and then to 100% B at 40 min (system 5). RP-TLC (Silica gel 60 RP-18 F254S, Merck, Tokyo, Japan) was developed with a mixture of 0.1% aqueous TFA and acetonitrile with 0.1% TFA (1:1, v/v). The radioactivity of fractions that RP-TLC was cut into 5 mm were measured with an auto-well gamma counter (Wizard 3). In-DOTA-GGNle-cycMSH_hex_ and [^111^In]In-DOTA-GGNle-cycMSH_hex_ were prepared by the same procedure described previously [19]. The RCCs were determined by RP-HPLC and partially also determined by RP-TLC. The RCYs were calculated from the obtained radioactivity and the initially used radioactivity and shown as the decay-corrected values.

# **Production of ^211^At**

^211^At was supplied from the National Institutes for Quantum Science and Technology and RIKEN through the Supply Platform of Short-lived Radioisotopes. ^211^At was produced by the ^209^Bi(α, 2n)^211^At reaction, and the detailed procedures were described in the previous reports [27, 33]. ^211^At was isolated from the irradiated target using the dry distillation method and eluted with chloroform_._ The solvent was dried by N_2_ gas, and a solid ^211^At was dissolved in acetonitrile.

# **Syntheses**


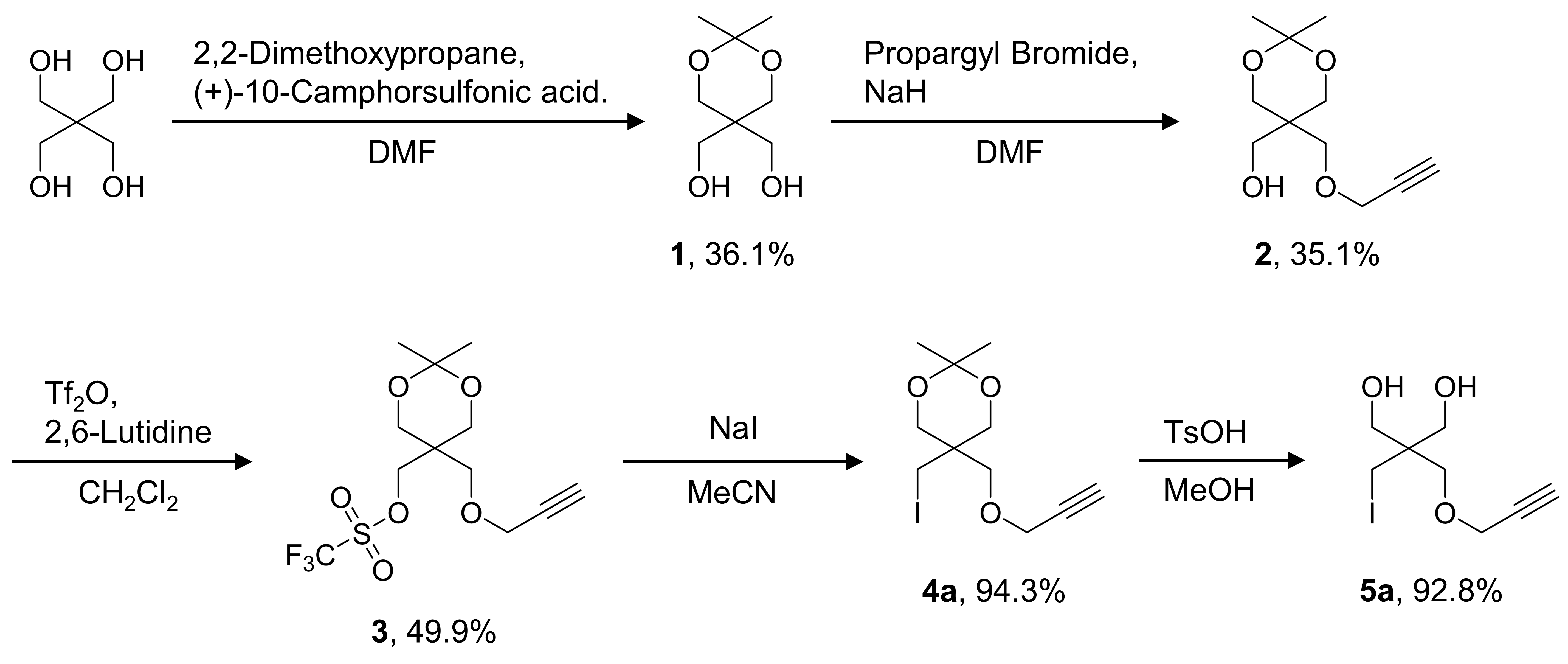


**Supplementary scheme 1.** Synthetic scheme of the precursor (**3**) for CuAAC reagent for astatination and the standard compound (**5a**) for charcterizing [^125^I]**5b** and [^211^At]**5c**.

## **(2,2-Dimethyl-1,3-dioxane-5,5-diyl)dimethanol (1)**

2,2-Dimethoxypropane (6.5 ml, 0.0528 mol) was added dropwise to a mixture of pentaerythritol (6.0 g, 0.044 mol) and (+)-10-camphorsulfonic acid (0.205 g, 0.883 mmol) in DMF (120 mL) at 40 °C. After cooling to room temperature, the mixture was stirred overnight. After triethylamine (TEA, 370 μL, 2.65 mmol) was added, the mixture was stirred for 10 min and then evaporated *in vacuo*. The crude compound was extracted from the residue by Soxhlet extraction method with hexane as a solvent. The extract was evaporated *in vacuo*, and hexane was added. The suspension was heated, and ethyl acetate was added dropwise until the mixture became a clear solution. After the mixture was stood overnight, the white precipitate was collected and dried *in vacuo* to provide **1** as a white solid (2.8 g, 0.0159 mol, 36.1%). Rf=0.52 (chloroform/methanol, 9/1). ^1^H-NMR (400MHz, CDCl_3_): δ 1.38 [6H, s, C*H_3_*], 3.57 [4H, s, C*H_2_*], 3.73 [4H, s, C*H_2_*] ppm. ^13^C NMR (150 MHz, CDCl_3_): δ 24.1 (2 C), 40.6, 62.7 (2 C), 63.3 (2 C), 99.3 ppm. HR-MS(ESI) calcd. for C_8_H_16_NaO_4_ [M+Na]^+^: m/z 199.09463, found 199.09457.

## **(2,2-dimethyl-5-((prop-2-yn-1-yloxy)methyl)-1,3-dioxan-5-yl)methanol (2)**

A solution of **1** (950 mg, 5.39 mmol) in DMF (10 mL) was added dropwise to a solution of 60% NaH oil (216 mg, 6.47 mmol) at 0 °C. After warmed to room temperature, 80% propargyl bromide in toluene (9.2 mol/L, 293 µL, 2.70 mmol) was added to the mixture, and then stirred for 2 h at the same temperature. After methanol (1 mL) was added, the mixture was evaporated *in vacuo*. 5 % citric acid (5 mL) was added to the mixture, and extracted with ethyl acetate (10 mL × 3). The combined organic phase was washed with saturated NaCl (10 mL) and dried with Na_2_SO_4_. After the solvent was removed *in vacuo*, the residue was purified by flash column chromatography on silica gel using a mixture of hexane/ethyl acetate (starting from 94/6 to 50/50) as an eluent to provide **2** as a yellow oil (406 mg, 1.89 mmol, 35.1 %). Rf=0.30 (hexane/ethyl acetate, 2/1). ^1^H-NMR (400MHz, CDCl_3_): δ 1.41 [6H, s, C*H_3_*], 1.66 [1H, br, O*H*], 2.45 [1H, t, *J* = 2.4 Hz, C*H*], 3.62 [2H, s, C*H_2_*], 3.68 [2H, s, C*H_2_*], 3.73 [2H, s, C*H_2_*], 3.73 [2H, s, C*H_2_*], 4.16 [2H, d, *J* = 2.4 Hz, C*H_2_*] ppm. ^13^C NMR (150 MHz, CDCl_3_): δ 23.4, 24.0, 38.9, 58.8, 62.8 (2 C), 64.4, 71.4, 74.8, 79.4, 98.5 ppm. HR-MS(ESI) calcd. for C_11_H_18_NaO_4_ [M+Na]^+^: m/z 237.11028, found 237.11050.

## **(2,2-dimethyl-5-((prop-2-yn-1-yloxy)methyl)-1,3-dioxan-5-yl)methyl trifluoromethanesulfonate (3)**

Trifluoromethanesulfonic anhydride (89 µL, 0.54 mmol) was added dropwise to a solution of **2** (58 mg, 0.27 mmol) and 2,6-lutidine (189 µL, 1.62 mmol) in CH_2_Cl_2_ (0.5 mL) at -78 °C. After warmed to -20 °C, the mixture was stirred overnight at the same temperature. After the mixture was warmed to room temperature, CHCl_3_ (5 mL) was added, and the combined organic phase was successively washed with saturated NaHCO_3_ (10 mL × 1), 5% citric acid (6 mL × 3), and saturated NaCl (5 mL × 1). After the organic phase was dried with Na_2_SO_4_, the solvent was removed *in vacuo*. The residue was purified by flash column chromatography on silica gel using a mixture of hexane/ethyl acetate (starting from 97/3 to 72/28) as an eluent to provide **3** as a yellow oil (46.8 mg, 0.135 mmol, 49.9 %). This compound was redissolved in diethyl ether (5–10 mg/mL) and stored at -80 °C. Rf=0.51 (hexane/ethyl acetate, 4/1). ^1^H-NMR (400MHz, CDCl_3_): δ 1.34 [3H, s, C*H_3_*], 1.37 [3H, s, C*H_3_*], 2.39 [1H, t, *J* = 2.4 Hz, C*H*], 3.37 [2H, s, C*H_2_*], 3.64 [2H, d, *J* = 12.8 Hz, C*H_2_*], 3.75 [2H, d, *J* = 12.4 Hz, C*H_2_*], 4.07 [2H, d, *J* = 2.4 Hz, C*H_2_*], 4.64 [2H, s, C*H_2_*], ppm. ^13^C NMR (150 MHz, CDCl_3_): δ 20.7, 26.3, 38.8, 58.7, 61.8 (2 C), 67.8, 75.2, 75.5, 78.8, 98.9, 118.6 (q, *J*_C-F_ = 317.85 Hz) ppm. HR-MS(ESI) calcd. for C_12_H_17_F_3_NaO_6_S [M+Na]^+^: m/z 369.05956, found 369.06082.

## **5-(iodomethyl)-2,2-dimethyl-5-((prop-2-yn-1-yloxy)methyl)-1,3-dioxane (4a)**

NaI (86.0 mg, 0.573 mmol) was added to a solution of **3** (99.3 mg, 0.287 mmol) in MeCN (1 mL). The mixture was stirred overnight at room temperature, and the solvent was removed *in vacuo*. After ethyl acetate (10 mL) was added to the residue, the organic phase was washed with water (10 mL × 3), and saturated NaCl (5 mL × 1). After the organic phase was dried with Na_2_SO_4_, the solvent was removed *in vacuo* to provide **4a** as a yellow oil (87.7 mg, 0.271 mmol, 94.3 %). Rf=0.65 (hexane/ethyl acetate, 4/1). ^1^H-NMR (400MHz, CDCl_3_): δ 1.41 [6H, s, C*H_3_*], 2.45 [1H, t, *J* = 2.6 Hz, C*H*], 3.36 [2H, s, C*H_2_*], 3.52 [2H, s, C*H_2_*], 3.70 [2H, d, *J* = 12.0 Hz, C*H_2_*], 3.80 [2H, d, *J* = 12.0 Hz, C*H_2_*], 4.16 [2H, d, *J* = 2.4 Hz, C*H_2_*] ppm. ^13^C NMR (150 MHz, CDCl_3_): δ 11.2, 23.5 (2 C), 36.9, 58.7, 64.7 (2 C), 70.8, 74.7, 79.4, 98.6 ppm. HR-MS(ESI) calcd. for C_11_H_17_INaO_3_ [M+Na]^+^: m/z 347.01201, found 347.01182.

## **2-(iodomethyl)-2-((prop-2-yn-1-yloxy)methyl)propane-1,3-diol (5a)**

*p*-Toulenesulfonic acid (69.9 mg, 0.406 mmol) was added to a solution of **4a** (87.7 mg, 0.271 mmol) in MeOH (1 mL). The mixture was stirred overnight at room temperature, and the solvent was removed *in vacuo*. After 5% NaHCO_3_ (0.4 mL) was added to the residue, the mixture was extracted with ethyl acetate (10 mL × 3). The combined organic phase was washed with saturated NaCl (10 mL × 1) and dried with Na_2_SO_4_. The solvent was removed *in vacuo* to provide **5a** as a yellow oil (71.3 mg, 0.251 mmol, 92.8 %). Rf=0.15 (hexane/ethyl acetate, 2/1). ^1^H-NMR (400MHz, CDCl_3_): δ 1.90 [2H, br, O*H*], 2.48 [1H, t, *J* = 2.2 Hz, C*H*], 3.32 [2H, s, C*H_2_*], 3.57 [2H, s, C*H_2_*], 3.68 [2H, d, *J* = 11.6 Hz, C*H_2_*], 3.74 [2H, d, *J* = 11.2 Hz, C*H_2_*], 4.17 [2H, d, *J* = 2.4 Hz, C*H_2_*] ppm. ^13^C NMR (150 MHz, CDCl_3_): δ 10.0, 43.5, 59.0, 65.1 (2 C), 71.9, 75.3, 79.4 ppm. HR-MS(ESI) calcd. for C_8_H_13_INaO_3_ [M+Na]^+^: m/z 306.98071, found 306.98053.

## **GGNle-CycMSH_hex_ analogs including an azido group**

Syntheses of GGNle-CycMSH_hex_ analogs were performed according to the procedure described previously with slight modifications [25]. Fmoc-protected amino acids used for the syntheses were Fmoc-Lys(Mtt)-OH, Fmoc-Trp(Boc)-OH, Fmoc-Arg(Pbf)-OH, Fmoc-D-Phe-OH, Fmoc-His(Trt)-OH, Fmoc-Asp(OPis)-OH, Fmoc-Nle-OH, Fmoc-Gly-OH, Fmoc-D-Glu(O^t^Bu)-OH, and Fmoc-D-Arg(Pbf)-OH. The peptides were manually extended from Rink-amide resin (200 mg, 0.142 mmol). Briefly, Fmoc-protected amino acids (2.5 equiv.), 1-hydroxybenzotriazole monohydrate (HOBt; 54.4 mg, 0.355 mmol, 2.5 equiv.) and *N,N’*-diisopropylcarbodiimide (DIC; 55 µL, 0.355 mmol, 2.5 equiv.) were added to a suspension of the resin in DMF (3 mL) and stirred using a tube rotator for 2 h. The Fmoc deprotection was performed by stirring with 20% v/v piperidine in DMF (3 mL) using a tube rotator for 20 min. The cyclization was conducted after the formation of Fmoc-Asp(OPis)-His(Trt)-D-Phe-Arg(Pbf)-Trp(Boc)-Lys(Mtt)-resin. After deprotection of Mtt and OPis groups by stirring with 2.5 % TFA/dichloromethane (3 mL), {{[(1-cyano-2-ethoxy-2-oxoethylidene)amino]oxy}-4-morpholinomethylene}dimethylammonium hexafluorophosphate (COMU; 182.4 mg, 3.0 equiv.) and DIEA (72.4 µL, 3.0 equiv.) were added to a suspension of the resin in DMF (3 mL) and stirred using a tube rotator overnight. Following elongation was performed by the same procedure described above to produce Fmoc-Gly-Gly-Nle-cyc(Asp-His(Trt)-D-Phe-Arg(Pbf)-Trp(Boc)-Lys)-resin (414 mg). This resin was divided into several portions, and each portion was used to synthesize N_3_-GGN**1 –** N_3_-GGN**4** by elongating the linkers and subsequently conjugating azidoacetic acid (2.5 equiv.) under the presence of HOBt (2.5 equiv.) and DIC (2.5 equiv.). After cleavage from resin and removal of protecting groups were performed by stirring with a cleavage cocktail (TFA:thioanisole:water:1,2-ethanedithiol:triisopropylsilane:phenol = 35/1/1/1/1/1, v/v/v/v/v/v) for 4 h, the mixture was filtered and evaporated *in vacuo*. Diethyl ether (2.0 mL) was added to the residue to give the precipitate. The precipitate was collected by filtration and purified by preparative RP-HPLC (system 4).

N_3_-GGN**1**. ESI-MS [M+H]^+^: m/z 1179.6, found 1179.6.
N_3_-GGN**2**. ESI-MS [M+H]^+^: m/z 1308.6, found 1308.6.

N_3_-GGN**3**. ESI-MS [M+H]^+^: m/z 1437.7, found 1437.7.

N_3_-GGN**4**. ESI-MS [M+H]^+^: m/z 1464.7, found 1464.7.

## **Non-radioactive iodinated GGNle-CycMSH_hex_ analogs**

Solutions of **5a** in the mixture of acetonitrile and water (1/4, v/v) (24 mM, 2.0 equiv.), tris[(1-benzyl-1*H*-1,2,3-triazol-4-yl)methyl]amine (TBTA) in DMSO (150 mM, 2.5 equiv.), CuSO_4_ in water (60 mM, 1.0 equiv.), and sodium L-ascorbate in water (150 mM, 2.5 equiv.) were added successively to the solution of GGNle-CycMSH_hex_ analogs including an azido group (N_3_-GGN**1** – N_3_-GGN**4**) in water (30 mM, 1.0 equiv.) with the volume ratio of 5/1/1/1/2 (**5a**/TBTA/CuSO_4_/sodium L-ascorbate/GGNle-CycMSH_hex_ analogs). The mixture was stirred overnight at 40 °C. After the reaction, the mixture was filtered through a 0.45 µm membrane filter (Millex-LH, Millipore). The filtrate was purified by preparative RP-HPLC (system 5) to provide non-radioactive iodinated GGNle-CycMSH_hex_ analogs as white solids.

NpG-GGN**1a**. ESI-MS [M+H]^+^: m/z 1463.6, found 1463.6.
NpG-GGN**2a**. ESI-MS [M+H]^+^: m/z 1592.6, found 1592.6.

NpG-GGN**3a**. ESI-MS [M+H]^+^: m/z 1721.7, found 1721.7.

NpG-GGN**4a**. ESI-MS [M+H]^+^: m/z 1748.7, found 1748.7.

# **References**

**19.** Guo H, Yang J, Gallazzi F, Miao Y. Effects of the Amino Acid Linkers on the Melanoma-Targeting and Pharmacokinetic Properties of ^111^In-Labeled Lactam Bridge–Cyclized α-MSH Peptides. J Nucl Med. 2011;52(4):608-16. <https://doi.org/10.2967/jnumed.110.086009>.

**25.** Zhang C, Zhang Z, Lin K-S, Pan J, Dude I, Hundal-Jabal N, et al. Preclinical melanoma imaging with ^68^Ga-labeled α-melanocyte-stimulating hormone derivatives using PET. Theranostics. 2017;7(4):805-13. <https://doi.org/10.7150/thno.17117>.

**27.** Watabe T, Kaneda-Nakashima K, Shirakami Y, Liu Y, Ooe K, Teramoto T, et al. Targeted alpha therapy using astatine (^211^At)-labeled phenylalanine: A preclinical study in glioma bearing mice. Oncotarget. 2020;11(15):1388-98. <https://doi.org/10.18632/oncotarget.27552>.

**33.** Ohshima Y, Sudo H, Watanabe S, Nagatsu K, Tsuji AB, Sakashita T, et al. Antitumor effects of radionuclide treatment using α-emitting *meta*-^211^At-astato-benzylguanidine in a PC12 pheochromocytoma model. Eur J Nucl Med Mol Imag. 2018;45:999-1010. <https://doi.org/10.1007/s00259-017-3919-6>.

# **Supplementary Tables**

**Supplementary Table 1.** Biodistribution of radioactivity 3 h after intravenous injection of ^125^I-labeled GGNle-CycMSH_hex_ analogs and [^111^In]In-DOTA-GGNle-cycMSH_hex_ into B16F10 tumor-bearing C57BL/6 mice.*^a^*

|  | [^125^I]NpG-GGN**1b** | [^125^I]NpG-GGN**2b** | [^125^I]NpG-GGN**3b** |
| --- | --- | --- | --- |
| Blood | 0.10 ± 0.04*^e^* | 0.09 ± 0.02*^e^* | 0.20 ± 0.04*^c,d,f,g^* |
| Liver | 3.86 ± 0.25*^d,e,f,g^* | 0.36 ± 0.28*^c,g^* | 0.15 ± 0.03*^c,f,g^* |
| Spleen | 0.14 ± 0.05*^g^* | 0.08 ± 0.08 *^f,g^* | 0.14 ± 0.05*^g^* |
| Kidney | 0.90 ± 0.64*^e,f,g^* | 0.75 ± 0.46*^e,f,g^* | 2.66 ± 0.48*^c,d,f,g^* |
| Pancreas | 0.09 ± 0.05 | 0.01 ± 0.02*^e,f^* | 0.09 ± 0.04*^d^* |
| Heart | 0.26 ± 0.38 | 0.12 ± 0.16 | 0.12 ± 0.03 |
| Lung | 0.31 ± 0.20 | 0.34 ± 0.13 | 0.26 ± 0.05 |
| Stomach*^b^* | 0.20 ± 0.20 | 0.20 ± 0.14*^f^* | 0.22 ± 0.19 |
| Intestine*^b^* | 22.91 ± 2.10*^d,e,f,g^* | 18.39 ± 2.39*^c,e,f,g^* | 1.06 ± 0.91*^c,d^* |
| Msucle | 0.11 ± 0.13 | 0.03 ± 0.03 | 0.20 ± 0.21 |
| Bone | 0.13 ± 0.04 | 0.11 ± 0.08 | 0.40 ± 0.27 |
| Thyroid*^b^* | 0.04 ± 0.01 | 0.02 ± 0.01 | 0.01 ± 0.00 |
| Tumor | 4.96 ± 0.55*^f,g^* | 3.76 ± 1.47*^e,f,g^* | 7.87 ± 2.69*^d,f,g^* |
|  | [^125^I]NpG-GGN**4b** | [^111^In]In-DOTA-GGNle-cycMSH_hex_ |  |
| Blood | 0.08 ± 0.02*^e^* | 0.08 ± 0.02*^e^* |  |
| Liver | 0.61 ± 0.07*^c,e^* | 0.76 ± 0.07*^c,d,e^* |  |
| Spleen | 0.26 ± 0.06*^d,g^* | 0.54 ± 0.09*^c,d,e,f^* |  |
| Kidney | 5.52 ± 1.01*^c,d,e^* | 4.99 ± 0.43*^c,d,e^* |  |
| Pancreas | 0.14 ± 0.03*^d^* | 0.07 ± 0.03 |  |
| Heart | 0.12 ± 0.06 | 0.05 ± 0.03 |  |
| Lung | 0.46 ± 0.21 | 0.22 ± 0.07 |  |
| Stomach*^b^* | 0.47 ± 0.13 | 0.20 ± 0.04 |  |
| Intestine*^b^* | 2.93 ± 1.06*^c,d^* | 0.60 ± 0.15*^c,d^* |  |
| Msucle | 0.12 ± 0.07 | 0.07 ± 0.02 |  |
| Bone | 0.19 ± 0.06 | 0.22 ± 0.07 |  |
| Thyroid*^b^* | 0.01 ± 0.00 | 0.01 ± 0.00 |  |
| Tumor | 13.47 ± 1.27*^c,d,e^* | 16.54 ± 1.89*^c,d,e^* |  |

*^a^*Data represent the mean of %ID/g ± SD (n = 4).

*^b^*Data represent the mean of %ID ± SD (n = 4).

*^c-f^P* < 0.05 compared with [^125^I]NpG-GGN**1b** (*^c^*), [^125^I]NpG-GGN**2b** (*^d^*), [^125^I]NpG-GGN**3b** (*^e^*), [^125^I]NpG-GGN**4b** (*^f^*), and [^111^In]In-DOTA-GGNle-cycMSH_hex_ (*^g^*). Significances were determined by one-way ANOVA followed by Tukey’s test.

**Supplementary Table 2.** Biodistribution of radioactivity 1, 3, 15 h after intravenous injection of [^125^I]NpG-GGN**4b** and [^211^At]NpG-GG**N4c** into B16F10 tumor-bearing C57BL/6 mice.*^a^*

|  | [^211^At]NpG-GGN**4c** | | | |
| --- | --- | --- | --- | --- |
|  | 1 h | 3 h | 15 h | 3 h (with blocking) |
| Blood | 1.13 ± 0.07 | 0.33 ± 0.02*^d^* | 0.15 ± 0.02 | 0.12 ± 0.03*^e^* |
| Liver | 1.45 ± 0.16 | 0.60 ± 0.03 | 0.24 ± 0.01*^c^* | 0.47 ± 0.12 |
| Spleen | 2.13 ± 0.46 | 1.11 ± 0.09*^d^* | 0.50 ± 0.14 | 0.20 ± 0.01*^e^* |
| Kidney | 24.20 ± 4.96*^c^* | 4.87 ± 0.31 | 0.70 ± 0.02*^c^* | 3.39 ± 0.85*^e^* |
| Pancreas | 0.62 ± 0.14 | 0.22 ± 0.04*^d,g^* | 0.12 ± 0.02*^c^* | 0.10 ± 0.04*^f^* |
| Heart | 1.25 ± 0.29*^c^* | 0.45 ± 0.05*^d,g^* | 0.15 ± 0.02*^c^* | 0.09 ± 0.06 *^f^* |
| Lung | 3.53 ± 0.77*^c^* | 1.43 ± 0.20 | 0.61 ± 0.08*^c^* | 0.55 ± 0.29 |
| Stomach*^b^* | 2.72 ± 1.28*^c^* | 1.50 ± 0.34*^d,g^* | 0.61 ± 0.04*^c^* | 0.07 ± 0.01*^e,f^* |
| Intestine*^b^* | 1.94 ± 0.83 | 1.94 ± 0.68 | 0.47 ± 0.14*^c^* | 1.64 ± 0.79*^e^* |
| Msucle | 0.31 ± 0.05 | 0.13 ± 0.05 | 0.02 ± 0.02*^c^* | 0.07 ± 0.05 |
| Bone | 0.95 ± 0.22 | 0.38 ± 0.05 | 0.14 ± 0.04*^c^* | 0.32 ± 0.25 |
| Thyroid*^b^* | 0.23 ± 0.06*^c^* | 0.12 ± 0.03*^d,g^* | 0.15 ± 0.03*^c^* | 0.00 ± 0.01*^f^* |
| Tumor | 12.96 ± 2.61 | 13.85 ± 2.23*^g^* | 2.72 ± 0.50 | 1.19 ± 0.18*^f^* |
|  | [^125^I]NpG-GGN**4b** | | | |
|  | 1 h | 3 h | 15 h | 3 h (with blocking) |
| Blood | 1.08 ± 0.07 | 0.15 ± 0.03*^f^* | 0.10 ± 0.06 | 0.13 ± 0.03*^g^* |
| Liver | 1.62 ± 0.15 | 0.56 ± 0.04 | 0.06 ± 0.01 | 0.50 ± 0.05 |
| Spleen | 1.92 ± 0.68 | 0.24 ± 0.04*^f^* | 0.32 ± 0.09 | 0.31 ± 0.08 *^g^* |
| Kidney | 32.00 ± 1.49 | 4.44 ± 0.35 | 0.24 ± 0.01 | 8.70 ± 1.35 *^g^* |
| Pancreas | 0.49 ± 0.04 | 0.08 ± 0.03*^f^* | 0.30 ± 0.04 | 0.14 ± 0.05 |
| Heart | 0.70 ± 0.11 | 0.09 ± 0.02*^f^* | 0.20 ± 0.03 | 0.12 ± 0.02 |
| Lung | 1.80 ± 0.24 | 0.69 ± 0.84 | 0.23 ± 0.05 | 0.31 ± 0.03 |
| Stomach*^b^* | 0.56 ± 0.16 | 0.65 ± 0.63*^f^* | 0.40 ± 0.12 | 0.48 ± 0.06 *^g^* |
| Intestine*^b^* | 1.02 ± 0.22 | 1.62 ± 1.32*^e^* | 0.02 ± 0.00 | 3.97 ± 1.49*^d,g^* |
| Msucle | 0.38 ± 0.06 | 0.06 ± 0.02 | 0.15 ± 0.02 | 0.11 ± 0.04 |
| Bone | 0.93 ± 0.09 | 0.29 ± 0.08 | 0.24 ± 0.02 | 0.25 ± 0.12 |
| Thyroid*^b^* | 0.07 ± 0.01 | 0.06 ± 0.01*^f^* | 0.07 ± 0.02 | 0.02 ± 0.01 |
| Tumor | 14.37 ± 4.03 | 12.83 ± 1.17*^e^* | 3.41 ± 0.33 | 0.89 ± 0.21*^d^* |

*^a^*Data represent the mean of %ID/g ± SD (n = 3-4).

*^b^*Data represent the mean of %ID ± SD (n = 3-4).

*^c^P* < 0.05 compared with [^125^I]NpG-GGN**4b** (*^c^*). Significances were determined by Student’s t-test.

*^d-g^P* < 0.05 compared with [^211^At]NpG-GGN**4c** without blocking (*^d^*), [^211^At]NpG-GGN**4c** with blocking (*^e^*), [^125^I]NpG-GGN**4b** without blocking (*^f^*), and [^125^I]NpG-GGN**4b** with blocking (*^g^*). Significances were determined by one-way ANOVA followed by Tukey’s test but not determined between [^211^At]NpG-GGN**4c** without blocking and [^125^I]NpG-GGN**4b** with blocking and between [^125^I]NpG-GGN**4b** without blocking and [^211^At]NpG-GGN**4c** with blocking.

# **Supplementary Figures**

## **Characterization and purity check for radiohalogenated compounds**

### **[^125^I]5b**

(A) Chromatogram of **5a**

**
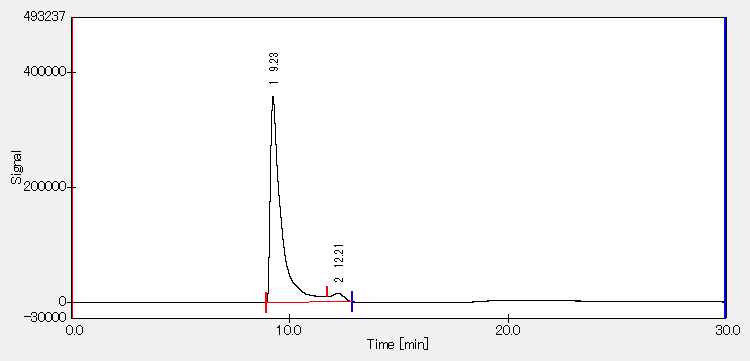
**

(B) Radiochromatogram of [^125^I]**5b** (RCP: >99%)


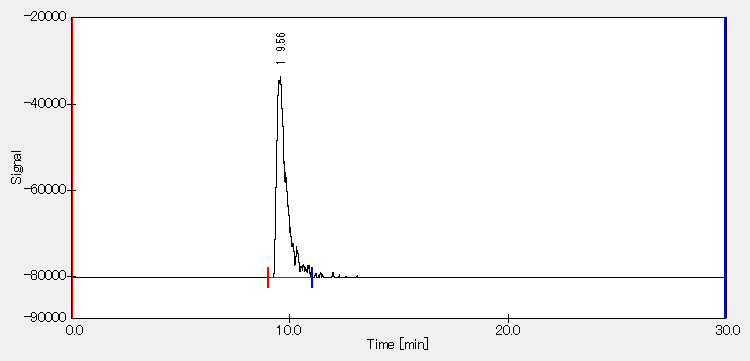


**Supplementary Fig. 1**. Characterization and purity check for [^125^I]**5b**.

### **[^211^At]5c**

(A) Chromatogram of **5a**

**
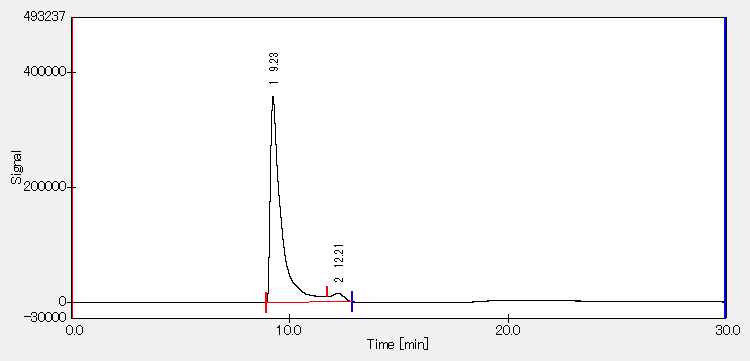
**

(B) Radiochromatogram of [^211^At]**5c** (RCP: >99%)


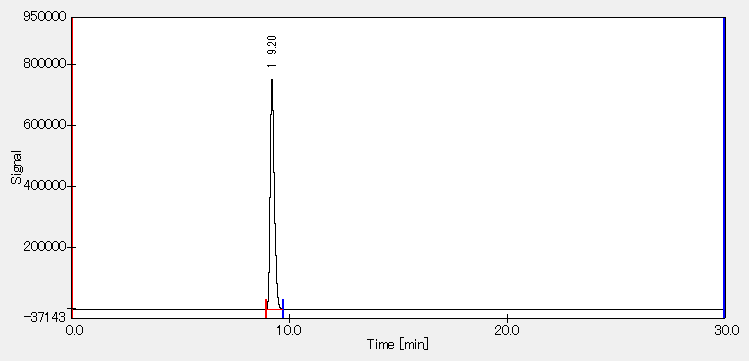


**Supplementary Fig. 2**. Characterization and purity check for [^211^At]**5c.**

### **[^125^I]NpG-GGN1b**

(A) Chromatogram of NpG-GGN**1a**


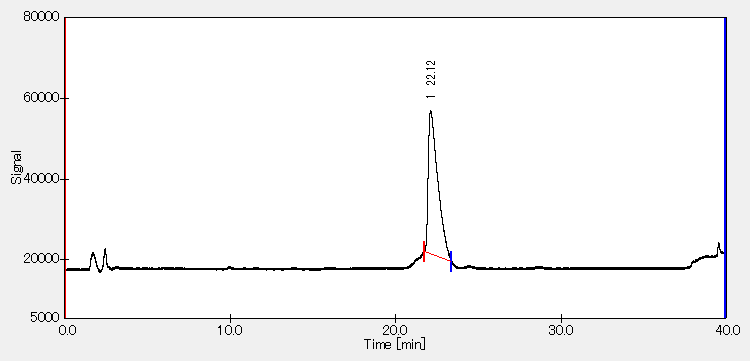


(B) [^125^I]NpG-GGN**1b** (RCP: >99%)


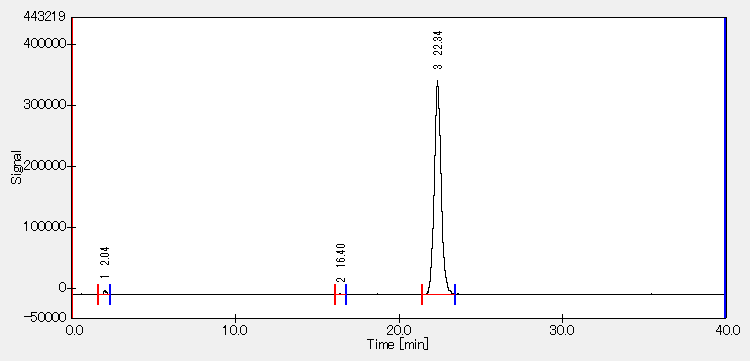


**Supplementary Fig. 3**. Characterization and purity check for [^125^I]NpG-GGN**1b**.

### **[^125^I]NpG-GGN2b**

(A) Chromatogram of NpG-GGN**2a**

**
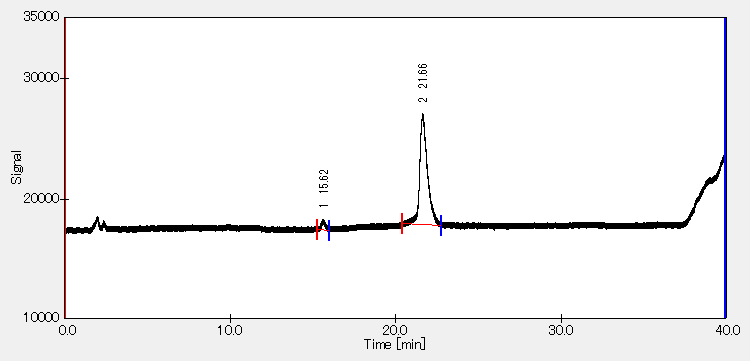
**

(B) [^125^I]NpG-GGN**2b** (RCP: >99%)


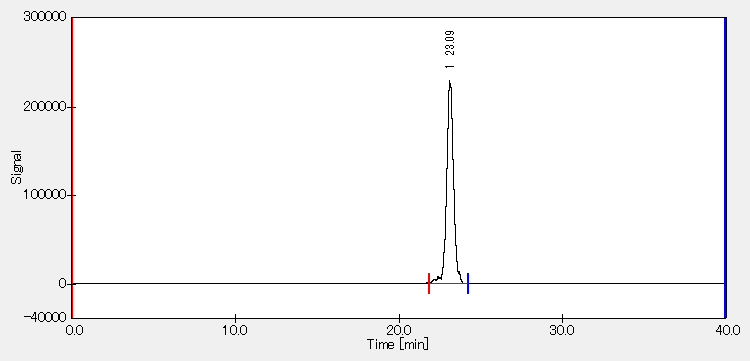


**Supplementary Fig. 4**. Characterization and purity check for [^125^I]NpG-GGN**2b**.

### **[^125^I]NpG-GGN3b**

(A) Chromatogram of NpG-GGN**3a**

**
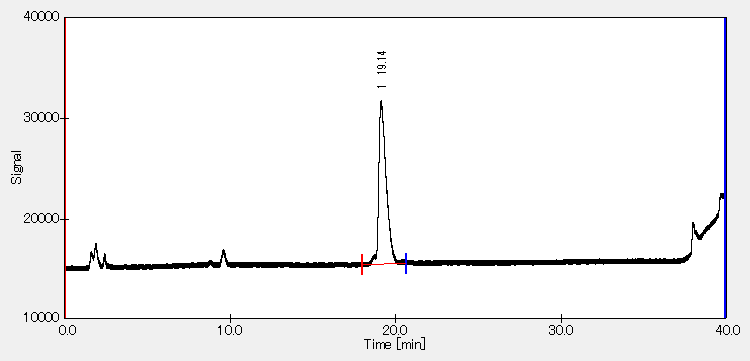
**

(B) [^125^I]NpG-GGN**3b** (RCP: 98.4%)


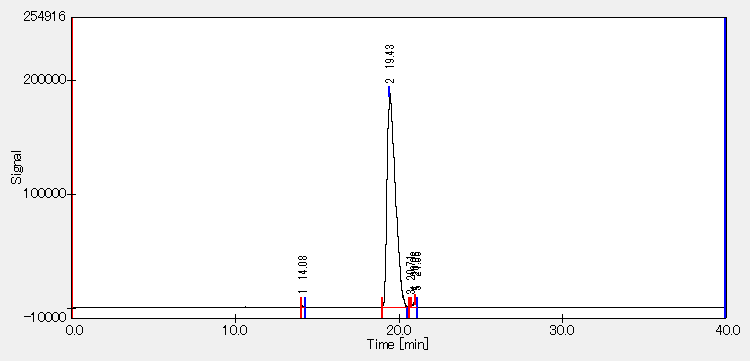


**Supplementary Fig. 5**. Characterization and purity check for [^125^I]NpG-GGN**3b**.

### **[^125^I]NpG-GGN4b**

(A) Chromatogram of NpG-GGN**4a**

**
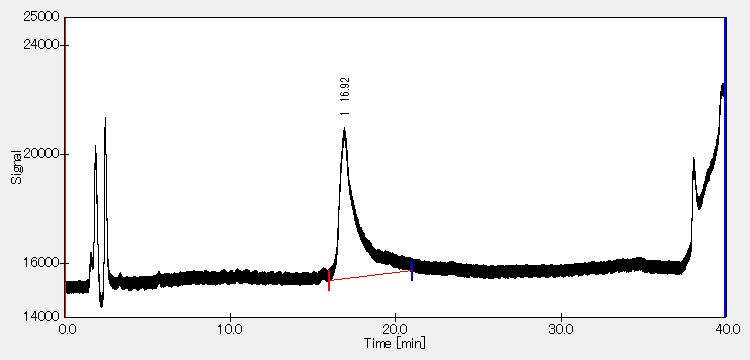
**

(B) [^125^I]NpG-GGN**4b** (RCP: >99%)


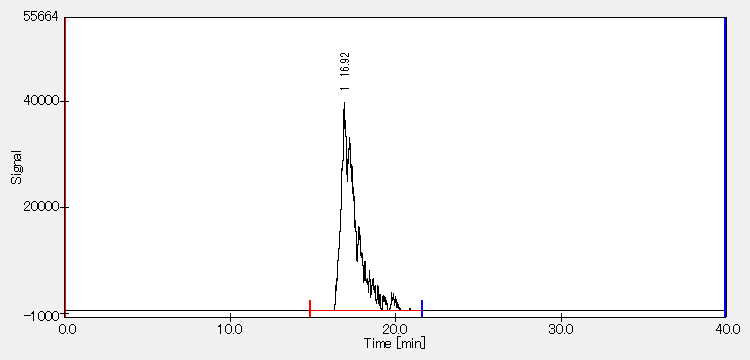


**Supplementary Fig. 6**. Characterization and purity check for [^125^I]NpG-GGN**4b**.

### **[^211^At]NpG-GGN4c**

(A) Chromatogram of NpG-GGN**4a**

**
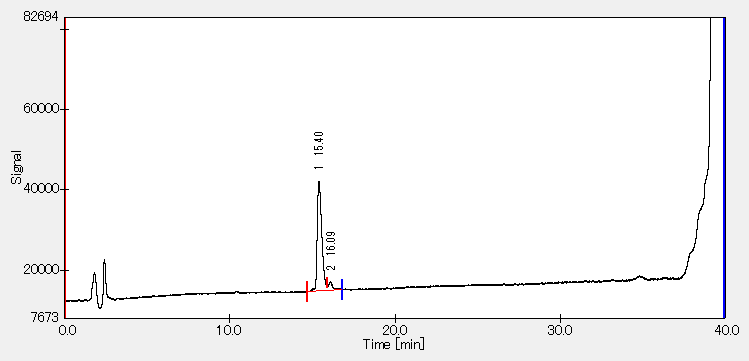
**

(B) [^211^At]NpG-GGN**4c** (RCP: >99%)


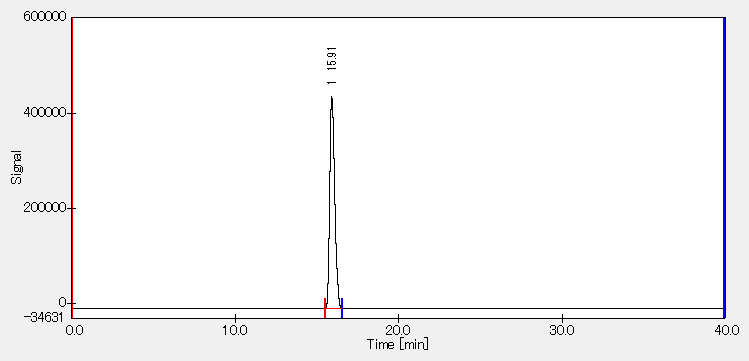


**Supplementary Fig. 7**. Characterization and purity check for [^211^At]NpG-GGN**4c**.

## **RP-TLC radiochromatograms for analyzing CuAAC reaction**


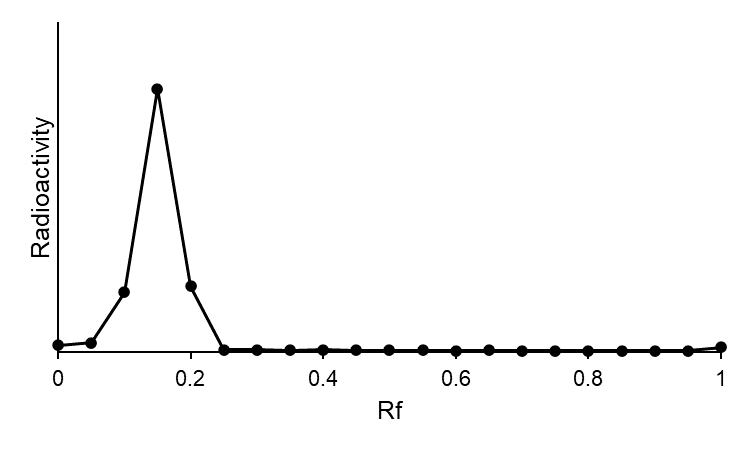


**Supplementary Fig. 8**. RP-TLC radiochromatogram for determining the RCC of the CuAAC reaction to produce [^211^At]NpG-GGN**4c** (RCC: 88.3%).

## ***In vitro* stability in murine plasma**

(A) Radiochromatograms before incubation in murine plasma

**
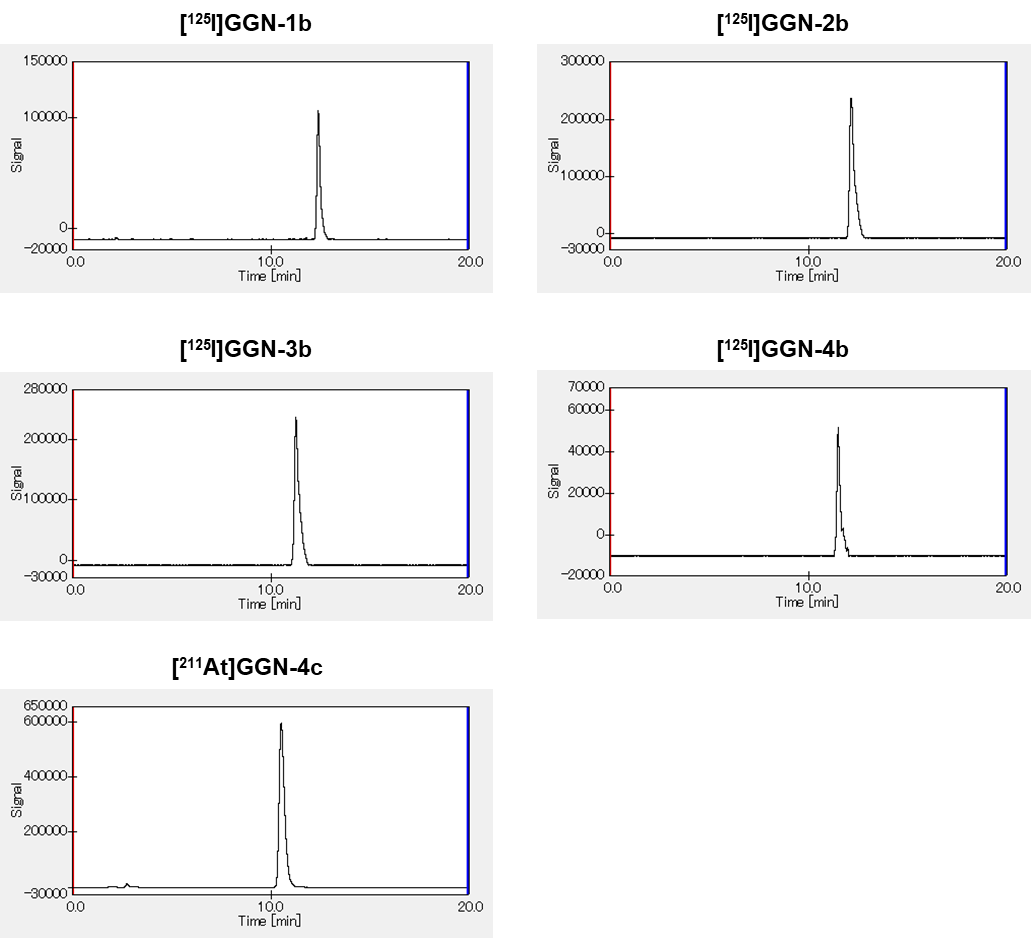
**

**(B)** Radiochromatograms after incubation in murine plasma

**
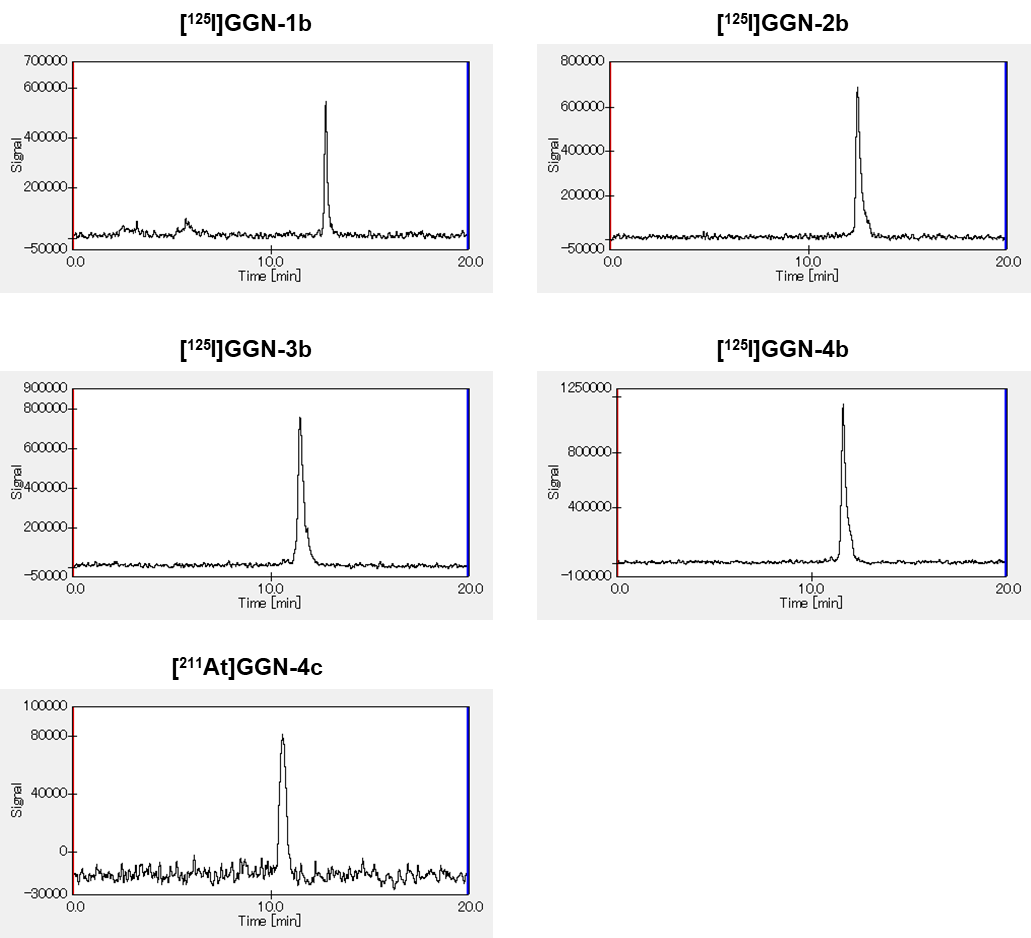
**

**Supplementary Fig. 9**. Typical RP-HPLC radiochromatograms of radiohalogenated GGNle-CycMSH_hex_ analogs (A) before and (B) after *in vitro* plasma stability experiments.

## **Urine analysis**

(A) Urine sample of [^211^At]NpG-GGN**4c**

**
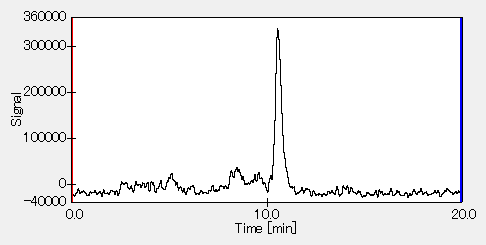
**

(B) Urine sample of [^125^I]NpG-GGN**4b**

**
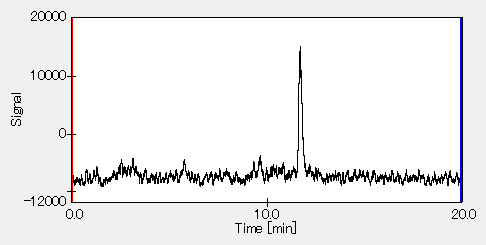
**

**Supplementary Fig. 10**. Typical RP-HPLC radiochromatograms analyzing the urine samples of (A) [^211^At]NpG-GGN**4c** and (B) [^125^I]NpG-GGN**4b**.

## **Therapeutic effect of [^211^At]NpG-GGN4c in individual mice**


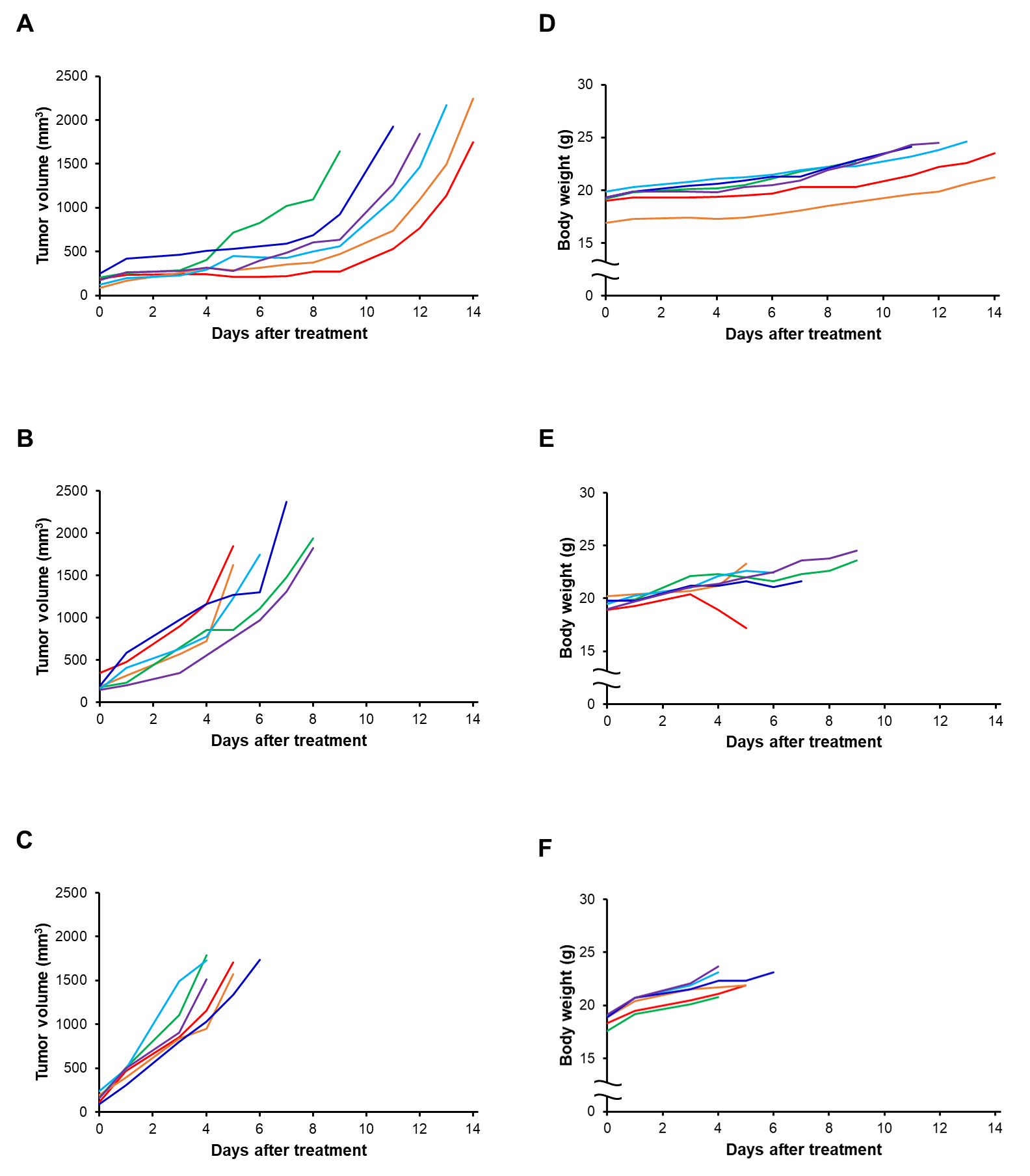


**Supplementary Fig. 11.** Therapeutic effect of [^211^At]NpG-GGN**4c** in individual B16F10 tumor-bearing mice. (A-C) Tumor volume in the groups treated with (A) 1 MBq of [^211^At]NpG-GGN**4c**; (B) 0.4 MBq of [^211^At]NpG-GGN**4c**; (C) saline. (D-F) Body weight in the groups treated with (D) 1 MBq of [^211^At]NpG-GGN**4c**; (E) 0.4 MBq of [^211^At]NpG-GGN**4c**; (F) saline.

# **MS spectra**

(A) GGNle-CycMSH_hex_ analogs including an azido group


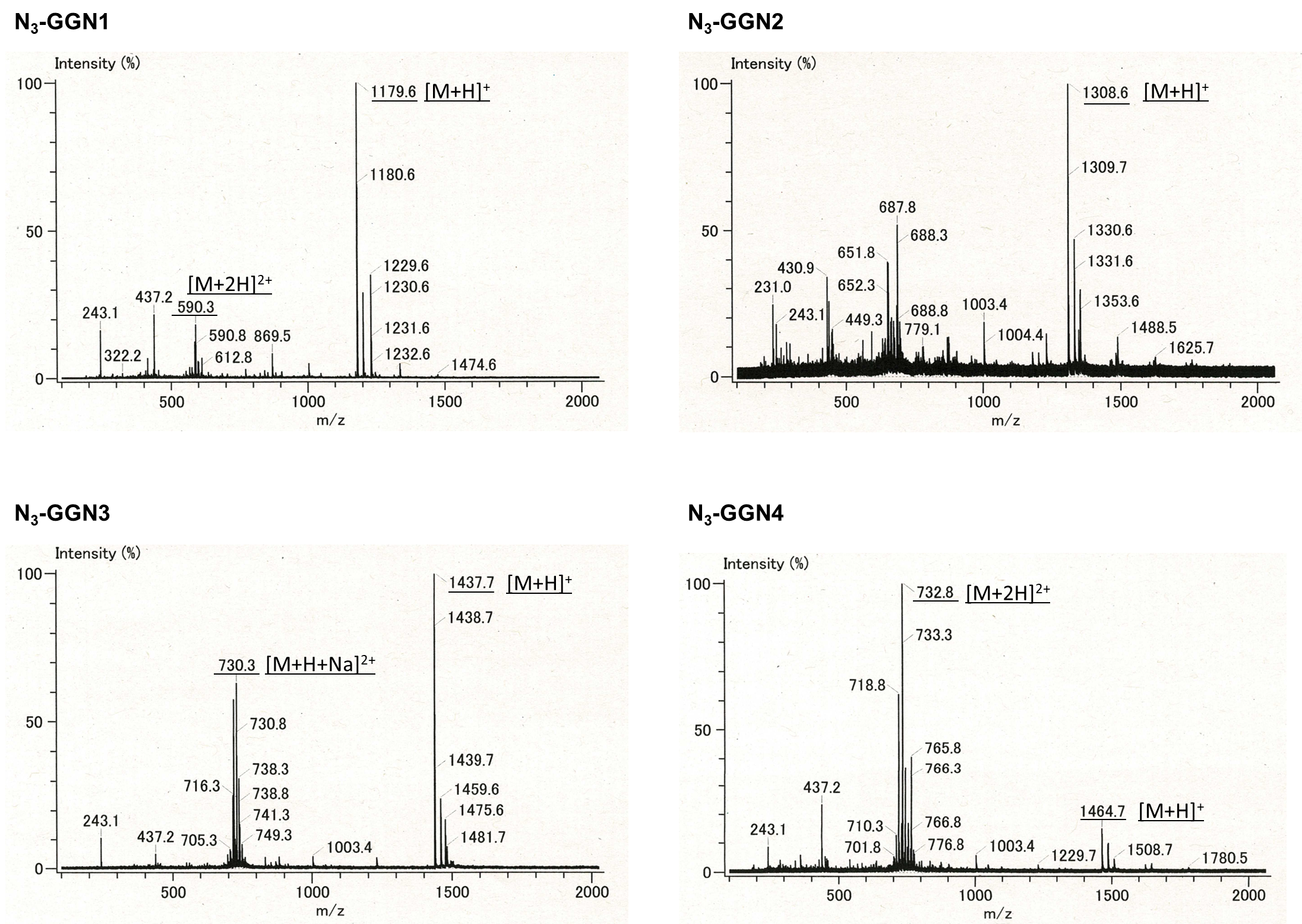


(B) Non-radioactive iodinated GGNle-CycMSH_hex_ analogs


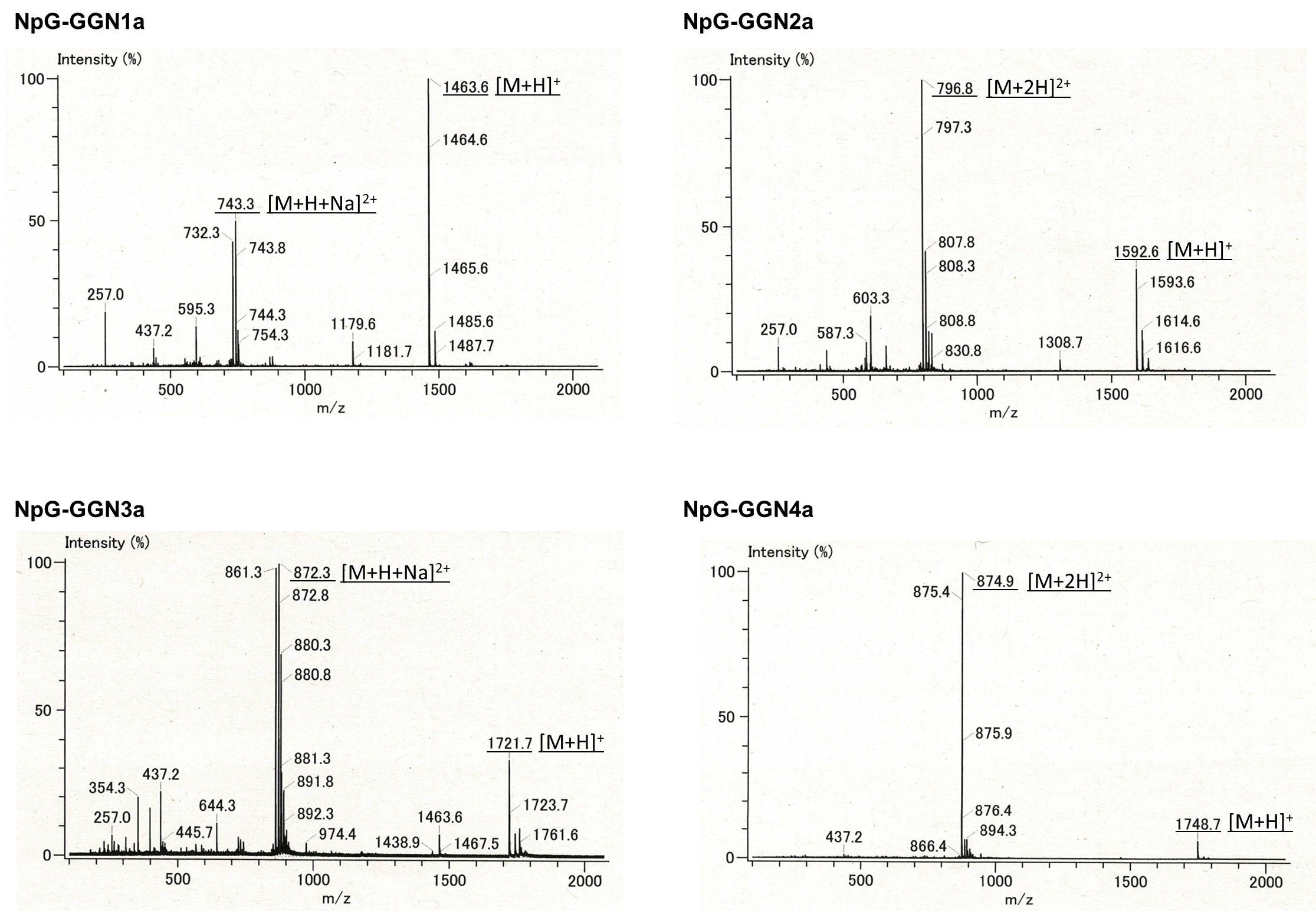


# **NMR Spectra**


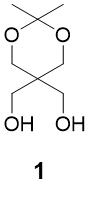

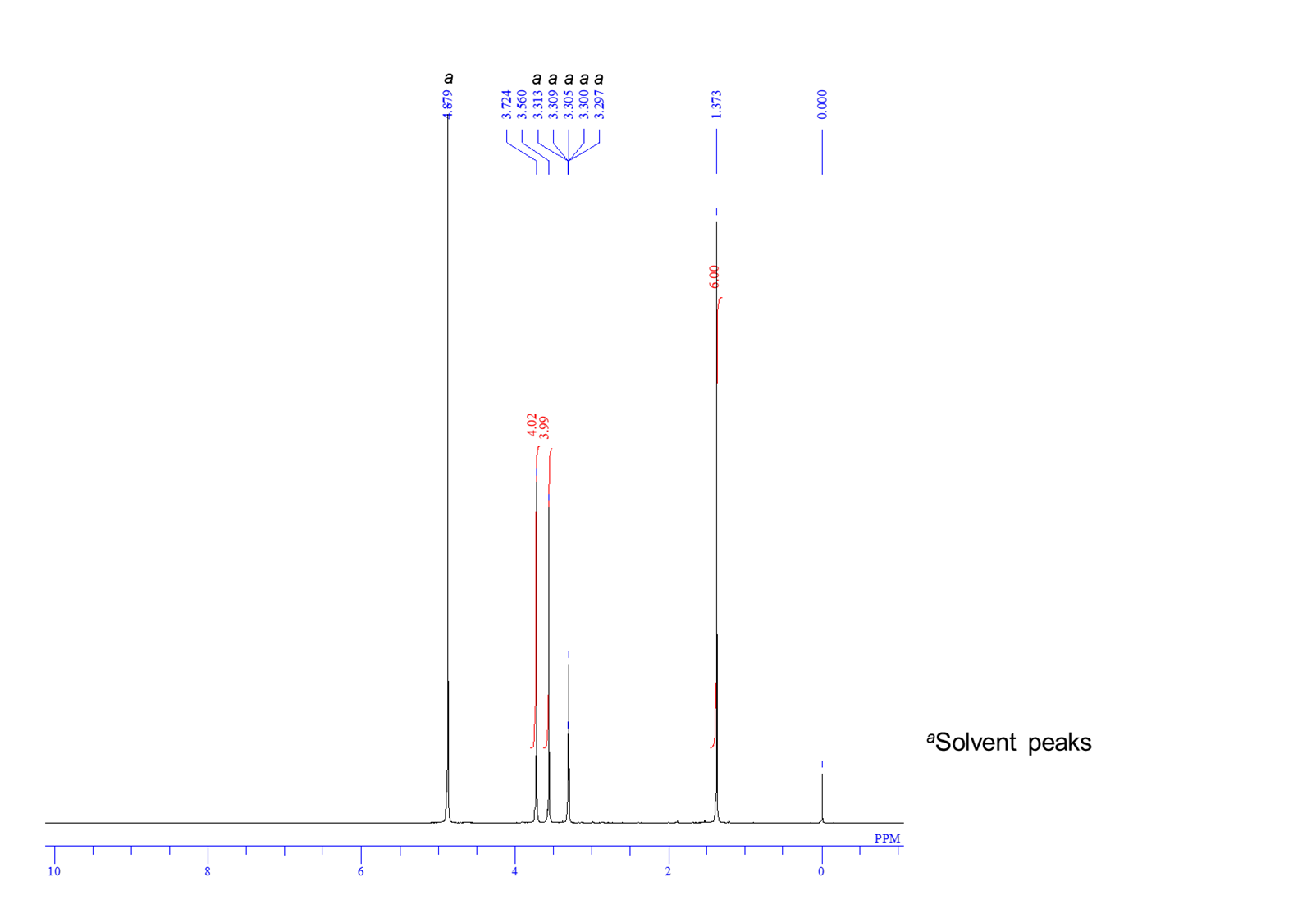


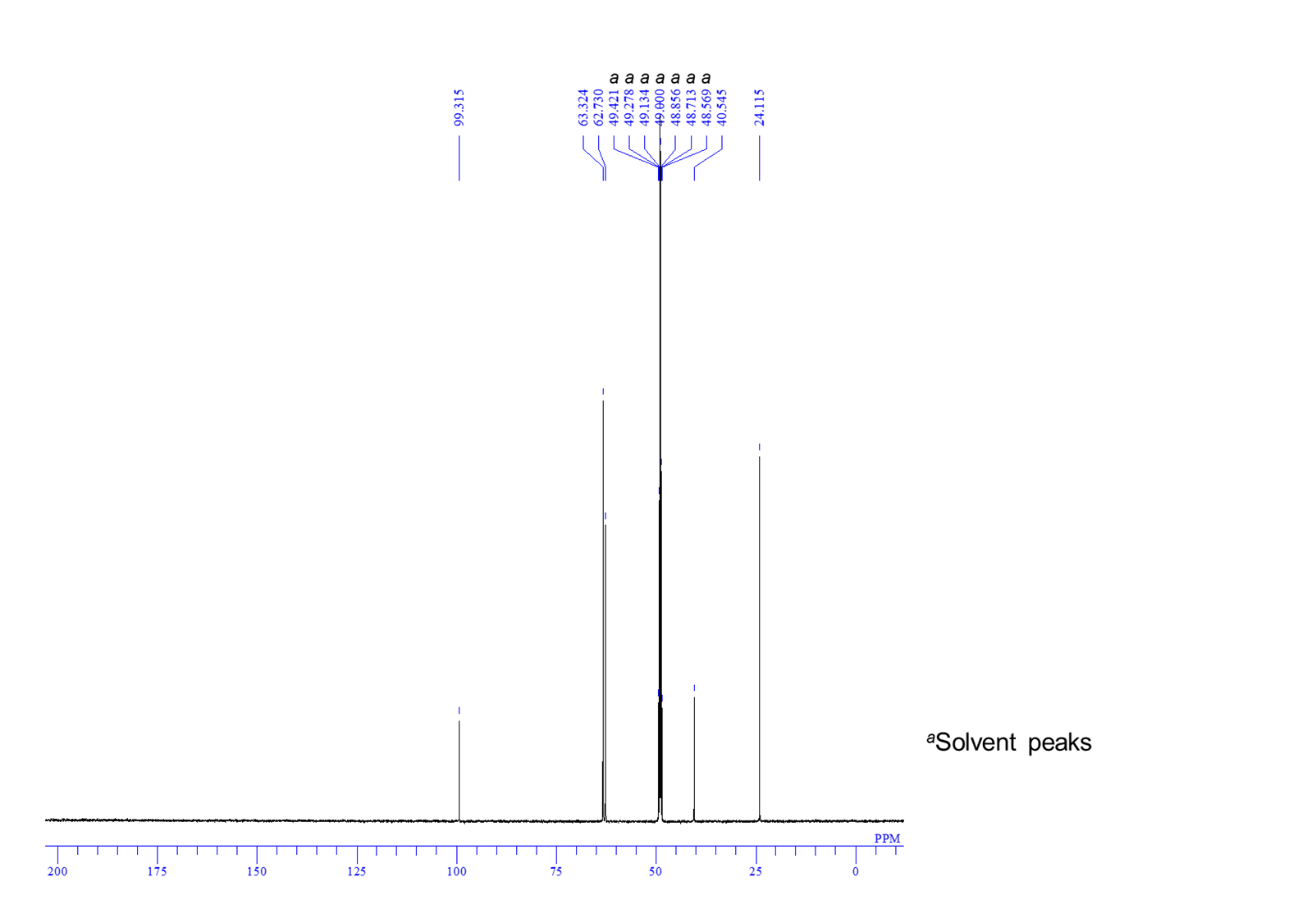


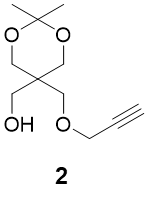

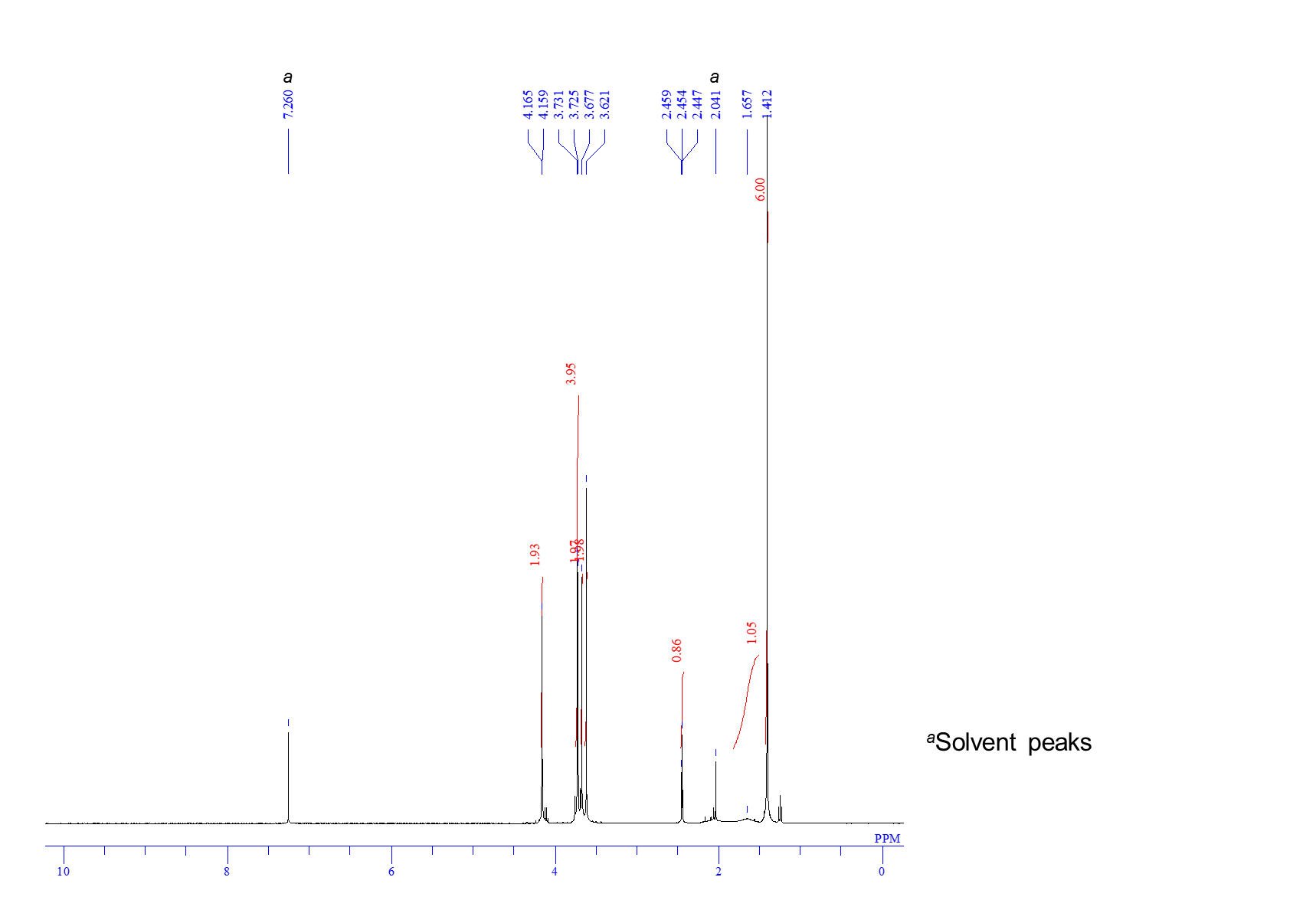

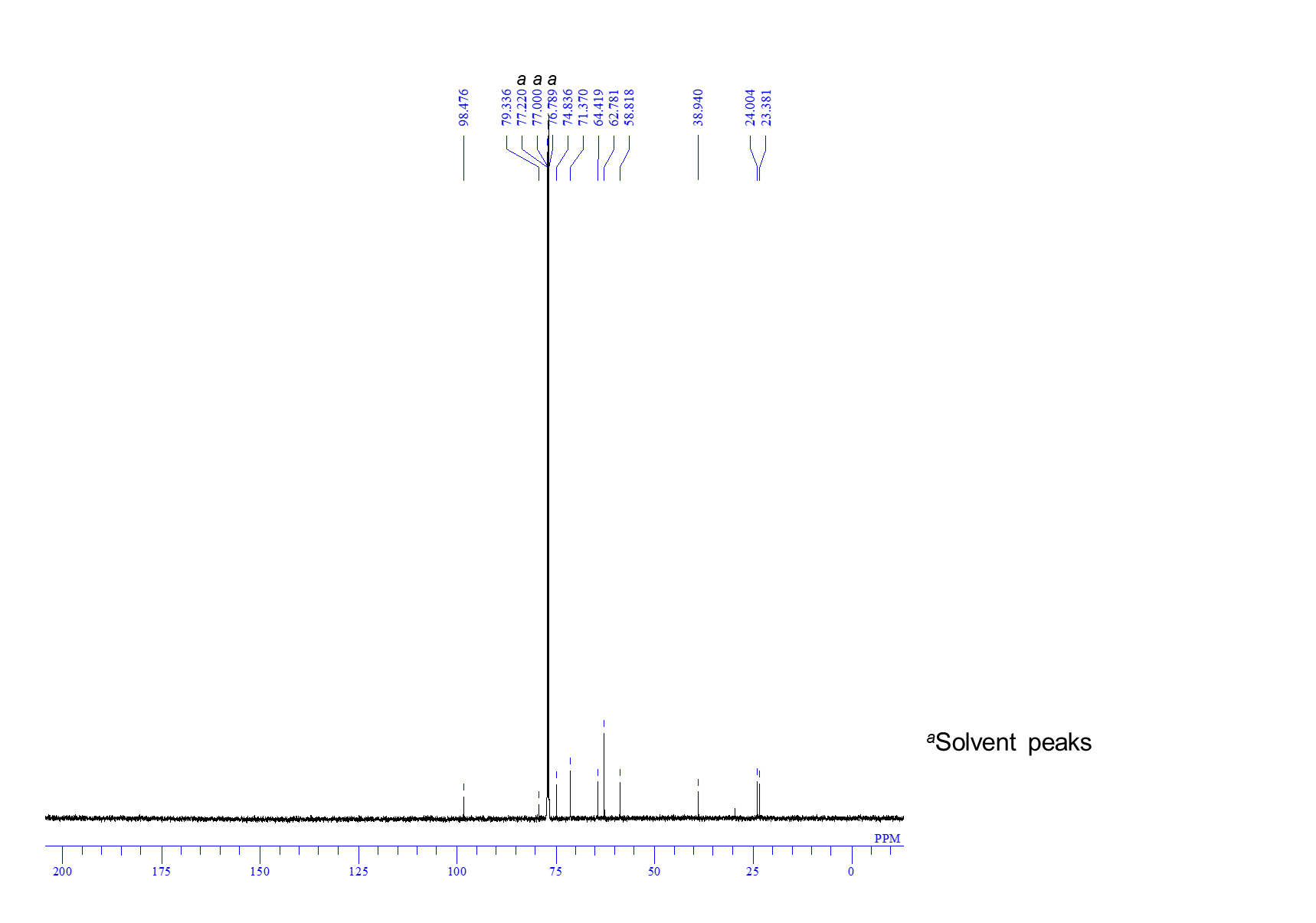


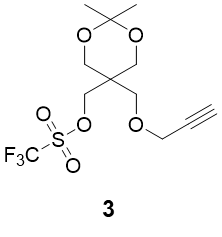

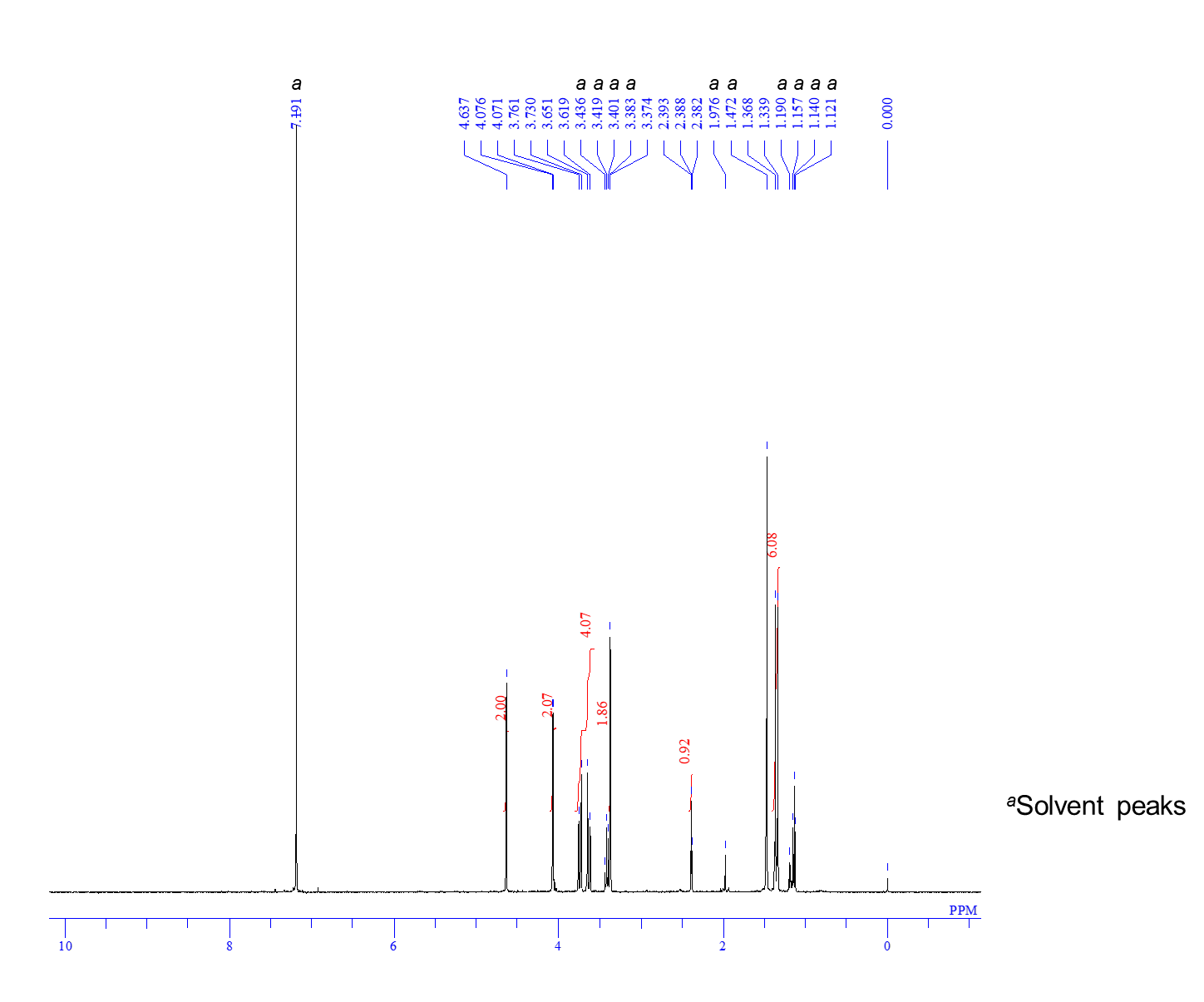


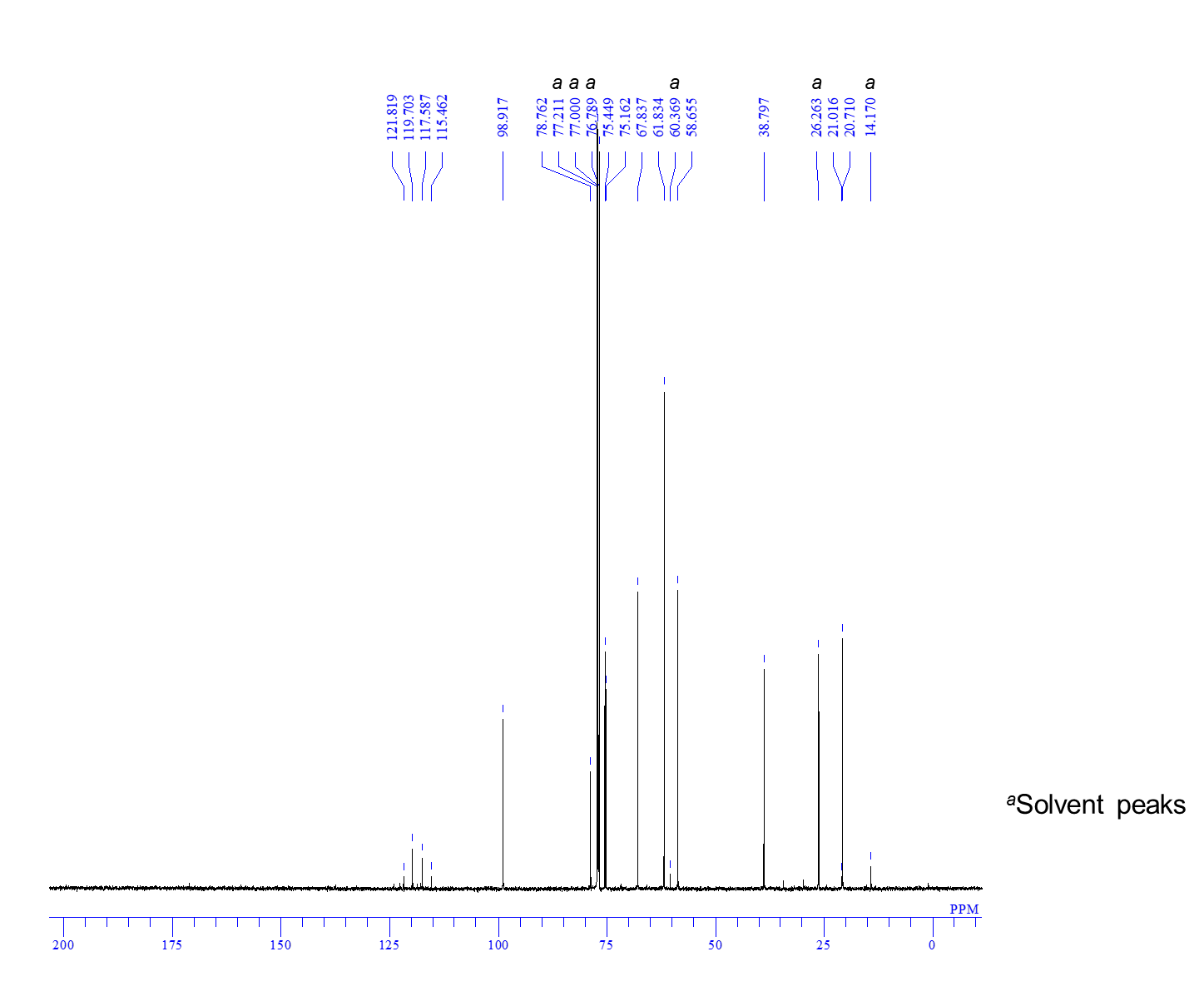


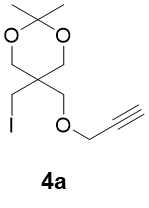

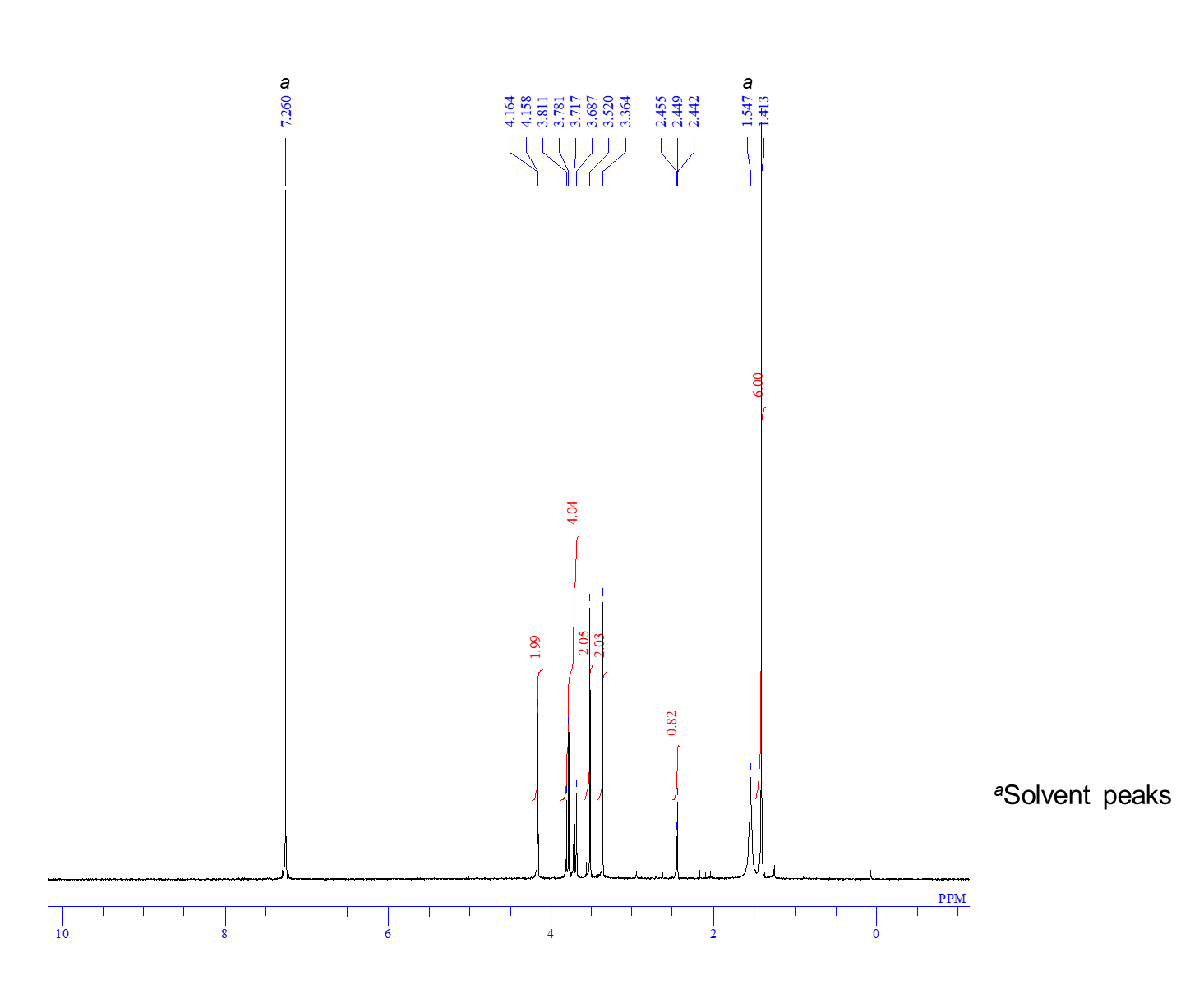


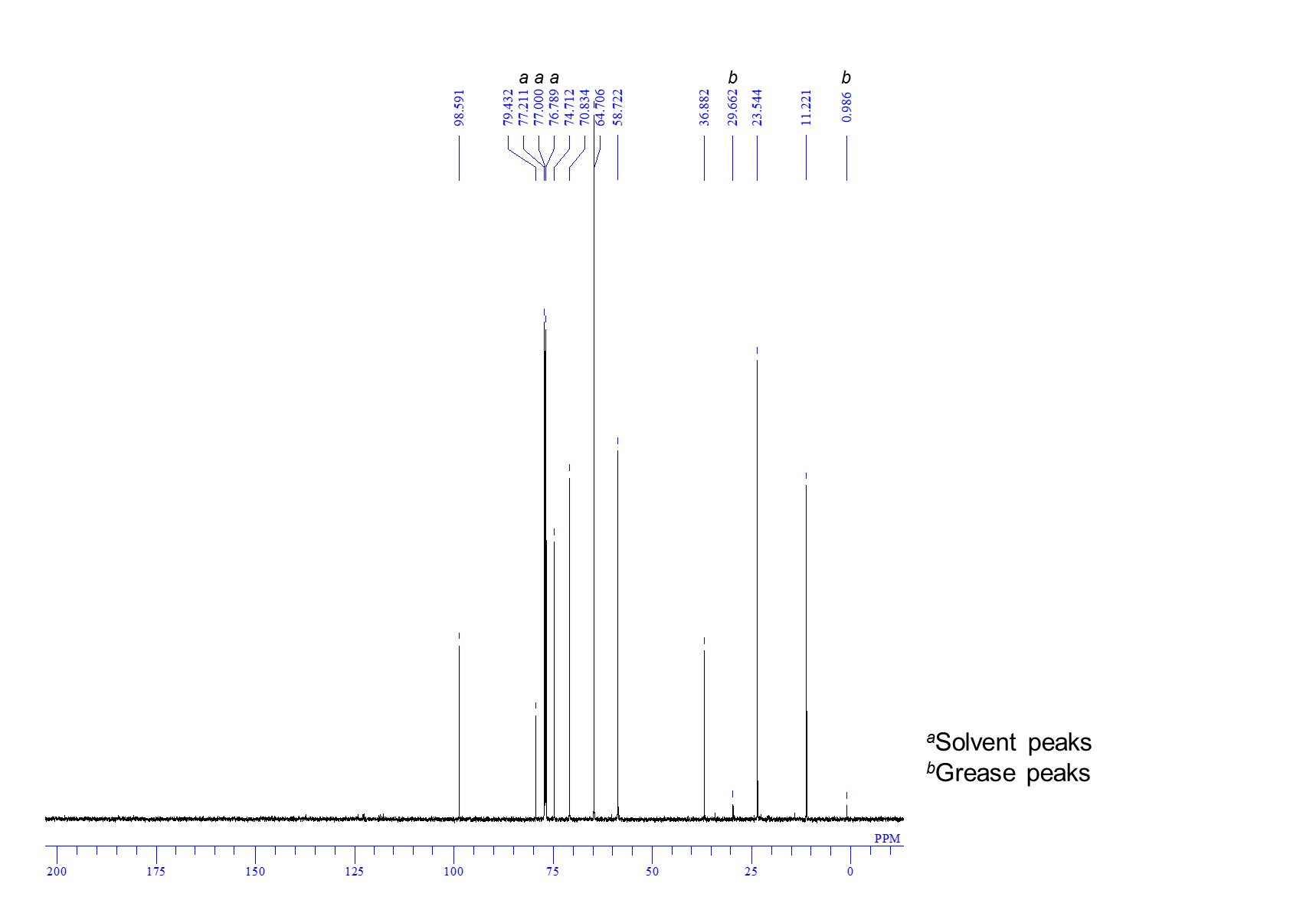


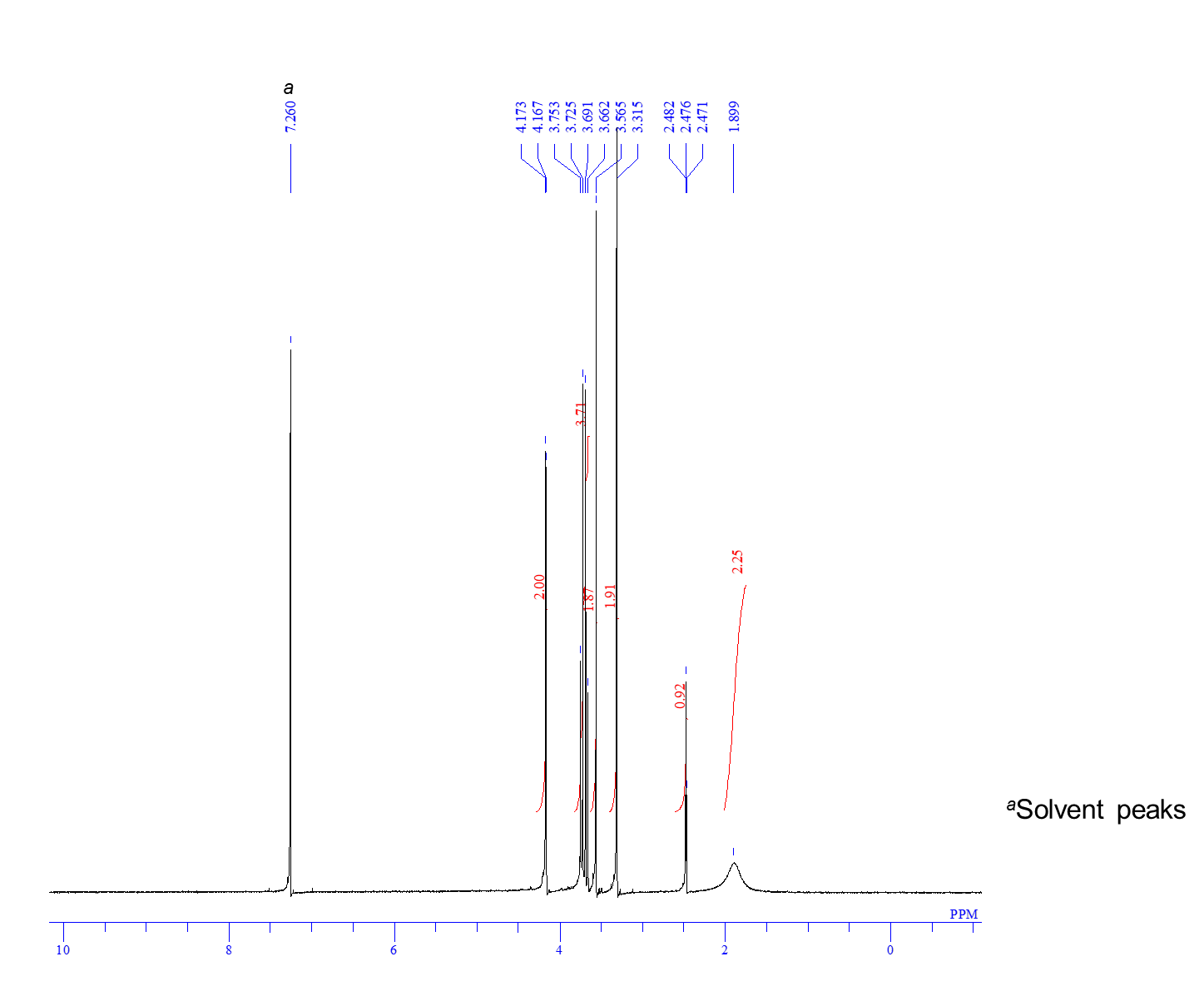

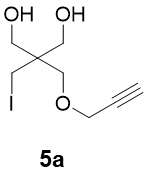


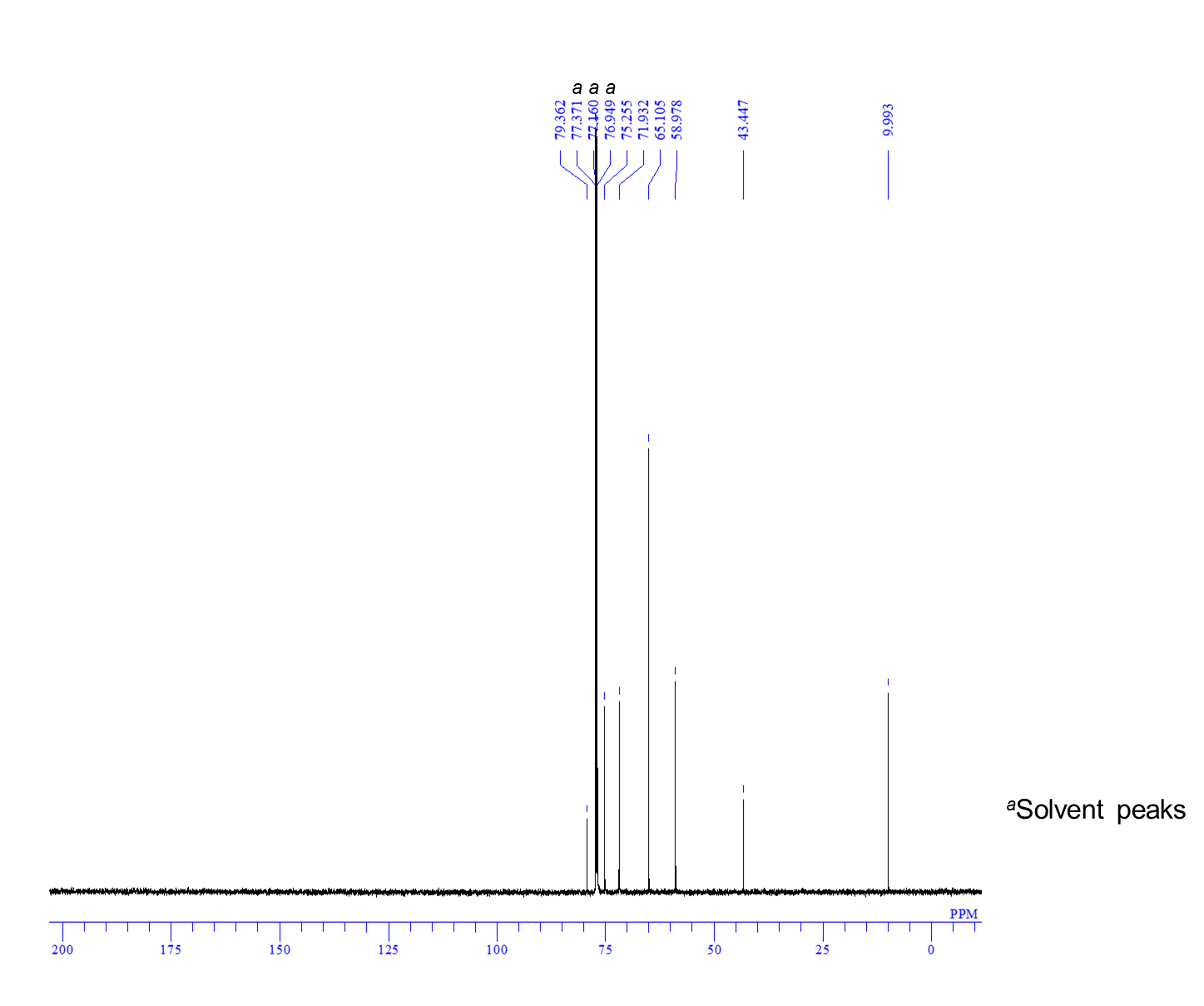

Supplement: Supplementary file 1 — (DOCX 8.21 MB) [file 259_2024_7056_MOESM1_ESM.docx]
